# Exploiting Genic Male Sterility in Rice: From Molecular Dissection to Breeding Applications

**DOI:** 10.3389/fpls.2021.629314

**Published:** 2021-03-02

**Authors:** Adil Abbas, Ping Yu, Lianping Sun, Zhengfu Yang, Daibo Chen, Shihua Cheng, Liyong Cao

**Affiliations:** ^1^Key Laboratory for Zhejiang Super Rice Research and State Key Laboratory of Rice Biology, China National Rice Research Institute, Hangzhou, China; ^2^State Key Laboratory of Subtropical Silviculture, Zhejiang A&F University, Hangzhou, China; ^3^Northern Center of China National Rice Research Institute, Shuangyashan, China

**Keywords:** genic male sterility, regulatory mechanism, anther and pollen development, biotechnology based male sterility systems, hybrid breeding, rice

## Abstract

Rice (*Oryza sativa* L.) occupies a very salient and indispensable status among cereal crops, as its vast production is used to feed nearly half of the world’s population. Male sterile plants are the fundamental breeding materials needed for specific propagation in order to meet the elevated current food demands. The development of the rice varieties with desired traits has become the ultimate need of the time. Genic male sterility is a predominant system that is vastly deployed and exploited for crop improvement. Hence, the identification of new genetic elements and the cognizance of the underlying regulatory networks affecting male sterility in rice are crucial to harness heterosis and ensure global food security. Over the years, a variety of genomics studies have uncovered numerous mechanisms regulating male sterility in rice, which provided a deeper and wider understanding on the complex molecular basis of anther and pollen development. The recent advances in genomics and the emergence of multiple biotechnological methods have revolutionized the field of rice breeding. In this review, we have briefly documented the recent evolution, exploration, and exploitation of genic male sterility to the improvement of rice crop production. Furthermore, this review describes future perspectives with focus on state-of-the-art developments in the engineering of male sterility to overcome issues associated with male sterility-mediated rice breeding to address the current challenges. Finally, we provide our perspectives on diversified studies regarding the identification and characterization of genic male sterility genes, the development of new biotechnology-based male sterility systems, and their integrated applications for hybrid rice breeding.

## Introduction

Rice is a major monoecious crop that has been successfully and extensively subjected to heterosis breeding through emasculation over time. Traditional methods of emasculation may cause hybrid seed impurity caused by an incomplete removal of fertile anthers from the female plant. Genic male sterility (GMS) has emerged as an ideal tool to accelerate hybrid breeding. Male sterility is generally characterized by the impairment of the male reproductive development as a result of underlying genetic causes and leads to the malformation of male gametes and/or pollen. In rice, GMS originates from mutations in the nuclear genome that are either insensitive or sensitive to environmental conditions (Chen and Liu, 2014; Viana et al., 2019). In the case of environment-sensitive GMS (EGMS), male gametogenesis is often vulnerable to different environmental conditions, including temperature (TGMS), photoperiod (PGMS), and humidity (HGMS) (Chen and Liu, 2014; Xue et al., 2018).

Hybrid vigor is directly related to the genetic diversity observed between species and has considerably contributed to an increase in agricultural yield as a result of the superior phenotypes of hybrid plants. Cytoplasmic male sterility (CMS) occurs via the interaction of mitochondrial and nuclear genes and has been used for the production of a three-line hybrid system. However, the poor genetic diversity of sterile parents and unreliable fertility restoration has restricted its use for breeding rice hybrids. Hence, GMS is an ideal replacement of CMS by overcoming these limitations through its stable sterility and safe hybrid production. Specifically, GMS has already assisted breeders to harness yield associated with hybrid vigor by artificially suppressing autogamy, and thus promoting allogamy, by the transmission of genes with favorable characteristics (Chen and Liu, 2014; Bohra et al., 2016, 2017). Hybrids have a higher fitness (due to heterosis) and produce up to 20% more yield than inbred rice varieties (Normile, 2008; Chen and Liu, 2014; Yuan, 2014), whereby GMS has become an invaluable resource to bring adaptive genetic diversity by facilitating the pollination of genetically diverse parental lines, a prerequisite to improve hybrid seed production.

The development of the anther and pollen is a complex genetic phenomenon that can easily be hampered and, therefore, requires a comprehensive understanding of the underlying molecular and biochemical mechanisms of male sterility to expand its scope for hybrid breeding (Bohra et al., 2016). The study of male sterility is an excellent way to critically understand the regulatory mechanisms that are essential for the complex male reproductive developmental process (Chen and Liu, 2014; Fan and Zhang, 2018). Several genomics studies have identified a number of GMS-related genes and analyzed the genetic, molecular, biochemical, and epigenetic pathways regulating anther and pollen development. In this review, we discuss the most recent developments and achievements regarding the identification of GMS genetic elements and their regulatory pathways in rice. Moreover, we present prospects for future strategies regarding the development of biotechnology-based male sterility systems and evaluate the application of these findings to efficiently break the genetic barriers of rice hybrid breeding.

## Genetic and Biochemical Regulatory Mechanisms of GMS Genes

**FIGURE 1:**
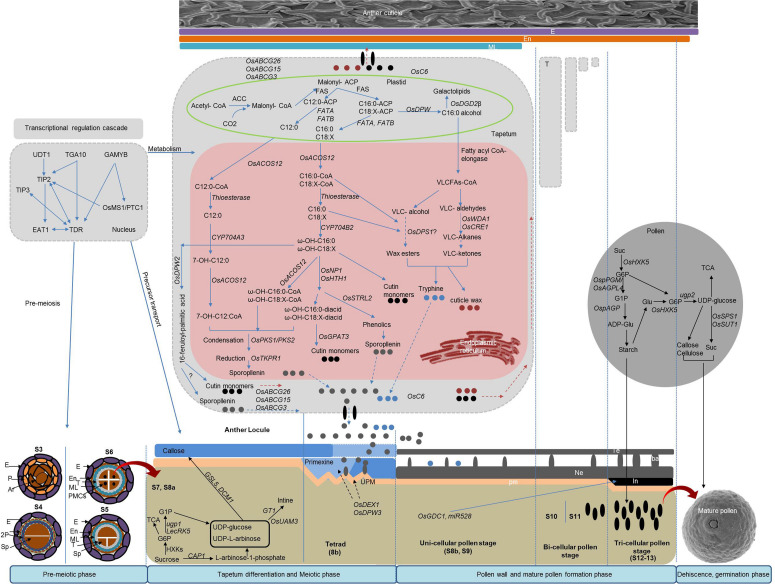
Genetic network of genic male sterility (GMS) genes for anther and pollen development including lipid and polysaccharide metabolism. E, epidermis; En, endothecium; ML, middle layer; T, tapetum; Sp, sporogenous cell; PMC, pollen mother cell; Ne, nexine; pm, plasma membrane; Te, tectum; ba, bacula; In, intine.

Anther development can be genetically and cytologically divided into 14 typical stages (Zhang et al., 2011). On the basis of cell division, differentiation, development, degradation, and maturation, we have divided the anther and pollen development process into four phases: (1) premeiotic phase, which includes archesporial and somatic cell specification, tapetum, and pollen mother cells (PMCs) specification; (2) tapetum differentiation and meiotic phase; (3) pollen wall and mature pollen formation phase; and (4) anther dehiscence and pollen germination phase (Figure 1). Cytological and biochemical analyses of different GMS mutants have reported numerous genes that are responsible for controlling anther and pollen development through transcriptional activity, metabolism, and other physiological processes, including anther dehiscence and pollen germination (Table 1).

**TABLE 1:** List of the characterized genes for genetic and biochemical regulation of anther and pollen development in rice.

RAP-ID	Gene	Encoding product	Biol. Function	Male sterility (MS)	References
Os07g0151100	*OsMILl*	CC-type glutaredoxin	Germline cells specifications	Complete MS	Hong et al., 2012b; Zhang Y.C. et al., 2020
Os09g0489500	*OsTGAlO*	bZIP transcription factor	Transcription factor	Partial MS	Chen et al., 2018
Os01g0917500	*OsMSPl*	Leucine-rich repeat receptor-like kinase	Sporogenic development, anther wall layer formation	Complete MS	Nonomura et al., 2003
Osl2g0472500	*OsMIL2*	Rice homolog of *AtTPD1*	Anther wall layers differentiation	Complete MS	Hong et al., 2012a
Os01g0293100	*TIP2*	bHLH DNA binding protein	Transcription factor	Complete MS	Fu et al., 2014; Ko et al., 2014
Os07g0549600	*UDT1*	bHLH DNA binding protein	Transcription factor	Complete MS	Jung et al., 2005
Os03g0296000	*TDF1*	R2R3 MYB transcription factor	Transcription factor	Complete MS	Cai et al., 2015
Os01g0812000	*GAMYB*	GAMYB transcrition factor	TF for GA pathway	Complete MS	Aya et al., 2009; Liu et al., 2010
Os01g0293100	*TDR*	bHLH transcription factor	Transcription factor	Complete MS	Li et al., 2006
Os04g0599300	*EAT1*	bHLH transcription factor	Transcription factor	Complete MS	Niu N. et al., 2013
Os04g0470600	*MYB80*	R2R3 MYB transcription factor	Transcription factor	Complete MS	Pan et al., 2020
Os07g0622900	*OsDTMl*	ER membrane protein	TA differentiation, PMC meiosis	Complete MS	Yi et al., 2012
Osl2g0443800	*OsFIGNLl*	AAA-ATPase	Meiosis	Complete MS	Zhang et al., 2017
Os09g0280600	*OsMFSl*	Meiotic coiled-coil protein	Meiosis	Complete MS	Lu et al., 2020
Os06g0182300	*OsGSL5*	Callose synthase	Callose biosynthesis	Complete MS	Shi et al., 2015c
Os06g0638000	*OsDCMl*	CCCH-tandem zinc finger protein	Callose biosynthesis	Complete MS	Zhang C. et al., 2020
Os02g0459600	*OsLecRK5*	Lectin receptor like kinase	Callose biosynthesis	Complete MS	Wang B. et al., 2020
Os09g0553200	*OsUGPl*	UDP-Glucose pyrophosphorylase 1	Callose biosynthesis	Complete MS	Chen et al., 2007
Os01g0947700	*OsGl*	Beta-1, 3-glucanase	Callose degradation	Complete MS	Wan et al., 2011
Os02g055000	*OsDMDl*	Nuclear protein	Callose degradation	Complete MS	Ren et al., 2020
Os07g0604800	*OsUAM3*	UDP-arabinopyranose mutase	Polysaccharide metabolism	Partial MS	Sumiyoshi et al., 2015
Os02g0141300	*OsCAPl*	Arabinokinase-Like Protein	Polysaccharide metabolism	Complete MS	Ueda et al., 2013
Os01g0262600	*OsGTl*	Glycosyltransferase	Polysaccharide metabolism	Partial MS	Moon et al., 2013
Os02g0100700	*OsDPW3*	Alpha integrin-like protein	Lipid metabolism, Callose biosynthesis	Complete MS	Mondol et al., 2020
Os03g0167600	*OsDPW*	fatty acyl-ACP reductase	Lipid metabolism	Complete MS	Shi et al., 2011
Os03g0268300	*OsDGD2β*	Digalactosyldiacylglycerol synthase	Lipid metabolism	Complete MS	Basnet et al., 2019
Os04g0310800	*OsACOS12*	Fatty acyl-CoA synthetase	Lipid metabolism	Complete MS	Li Y. et al., 2016
Osl0g0471100	*OsWDAl*	Aldehyde decarbonylase	Lipid metabolism	Complete MS	Jung et al., 2006
Os02g0621300	*OsCERl*	Aldehyde decarbonylase	Lipid metabolism	Complete MS	Ni et al., 2018
Os05g0395300	*OsDPSl*	Cystathionine b-synthase domain containing protein	Lipid metabolism	Complete MS	Zafar S.A. et al., 2020
Os03g0168600	*OsCYP704B2*	Cytochrome P450 protein	Lipid metabolism	Complete MS	Li et al., 2010
Os04g0573900	Os*CYP704A3*	Cytochrome P450 protein	Lipid metabolism	Complete MS	Aya et al., 2009; Yang et al., 2014, 2018
Osl0g0524500	*OsNPl*	Glucose-methanol-choline oxidoreductase	Lipid metabolism	Complete MS	Chang et al., 2016a; Liu Z. et al., 2017
Os04g0573100	OsHTHl	Glucose-methanol-choline oxidoreductase	Lipid metabolism	Complete MS	Xu Y. et al., 2017
Os03g0263600	*OsSTRL2*	Atypical strictosidine synthase	Lipid metabolism	Complete MS	Zou et al., 2017a
Osllg0679700	*OsGPAT3*	Glycerol-3 -phosphate acyltransferase	Lipid metabolism	Complete MS	Men et al., 2017; Sun et al., 2019
Os01g0924933	*OsDPW2*	BAHD acyltransferase	Lipid metabolism	Complete MS	Xu D. et al., 2017
Osl0g0484800	*OsPKSl*	Chalcone and stilbene synthase	Lipid metabolism	Complete MS	Zou et al., 2017b
Os07g0411300	*OsPKS2*	Chalcone and stilbene synthase	Lipid metabolism	Complete MS	Zou et al., 2018
Os09g0493500	*OsTKPRl*	Tetraketide a-pyrone reductase	Lipid metabolism	Complete MS	Xu et al., 2019
Osllg0582500	*OsC6*	Non-specific lipid transfer protein C6	Lipid metabolism	Partial MS	Zhang D. et al., 2010
Os01g083600	*OsABCG3*	ATP-binding cassette (ABC) transporter G3	Lipid metabolism	Complete MS	Chang et al., 2018; Luo et al., 2019
Os06g0607700	*OsABCG15*	ATP-binding cassette (ABC) transporter G15	Lipid metabolism	Complete MS	Niu B.X. et al., 2013; Wu et al., 2014
Osl0g0494300	*OsABCG26*	ATP-binding cassette (ABC) transporter G26	Lipid metabolism	Complete MS	Chang et al., 2016b
Os03g0825700	*OsDEXl*	Ca^2+^ binding protein	Lipid metabolism (Intine formation)	Complete MS	Yu et al., 2016
Os01g0801700	*OsGCDl*	Homolog of *AtGCD1*	Polysaccharide metabolism (Intine)	Complete MS	Huang et al., 2018, 2019
Os08g0137400	*OsUCL23*	Uclacyanin like protein 23	Flavonoid Metabolism (Intine)	Partial MS	Zhang Y.C. et al., 2020
Os03g0709100	*OsUCLS*	Uclacyanin like protein 8	Polysaccharide metabolism (Intine)	Partial MS	Zhang F. et al., 2018
Os05g0522500	*OsHXK5*	Hexokinase	Starch synthesis in pollen	Complete MS	Lee et al., 2020
Os10g0189100	*OspPGM*	Plastidic phosphoglucomutase	Starch synthesis in pollen	Complete MS	Lee et al., 2016
Os02g0117700	*OsUGP2*	UDP-Glucose pyrophosphorylase2	Starch synthesis in pollen	Partial MS	Mu et al., 2009
Os05g0586200	*OsJARl*	Jasmonyl-L-isoleucine synthase	JA biosynthesis, (Anther dehiscence)	Complete MS	Xiao et al., 2014
Os05g0370600	*OsFTIP7*	C2 domain and transmembrane region proteins	Auxin biosynthesis (Anther dehiscence)	Complete MS	Song et al., 2018
Os01g0293100	*bHLH142*	bHLH transcription factor	Anther dehiscence	Complete MS	Ranjan et al., 2017
Os01g0919400	*OsSPSl*	Sucrose phosphate synthase	Sucrose synthesis	Complete MS	Hirose et al., 2014
Os03g0170900	*OsSUTl*	Sucrose transporter protein	Apoplastic sucrose loading	Complete MS	Hirose et al., 2010
Os02g0661300	*OsINPl*	Homolog of *AtINP1*	Pollen aperture formation	Complete MS	Zhang Y.C. et al., 2020

*TA, tapetum; PMC: pollen mother cells; TF, transcription factor; GA, gibberellic acid; Biol. function, biological function.*

### Premeiotic Phase

#### Archesporial Cell Specification

The plant germline cells are the precursors of gamete production and generally originate from archesporial (AR) cells. Hence, any disruption during AR specification can lead to abnormal gamete development and thus male sterility.

Currently, very little is known about the GMS genes that are involved in this process in rice, although some examples have been described. *MICROSPORELESS1* (*OsMIL1*) encoding CC-type glutaredoxin is a germline-specific gene that controls the production of germ cells. Mutations in this gene are known to result in failure during differentiation into AR cells. Furthermore, molecular and cytological studies demonstrated that the anthers of *mil1* mutants fail to produce microspores and enter the meiotic phase (Hong et al., 2012b). Another example is the *OsTGA10* gene, which encodes a bZIP transcription factor and plays multiple roles such as determining cell specificity during early stamen development, tapetum, and microspore development. The mutants of *TGA10* show higher expression during earlier anther developmental stages (S2-S4) and cause abnormal endothecium and tapetum development, which indicates its role during early anther development (Chen et al., 2018). The interaction between the genes *TGA10* and *MIL1* promotes the next step of anther cell specification (Hong et al., 2012b).

#### Specification of Somatic and Sporogenous Cells

After the specification of AR cells, several genes are involved in controlling the development of somatic cell layers and the specification of sporogenous cells. *MULTIPLE SPOROCYTE 1* (*OsMSP1*) encodes a Leucine-rich repeat receptor-like kinase protein that is responsible for controlling the number of male and female cells during gametogenesis. Moreover, *msp1* mutants produce an excessive number of male and female sporogenous cells and exhibit a complete absence of the tapetum (Nonomura et al., 2003), while the gene *MICROSPORELESS 2* (*OsMIL2*) controls the differentiation of primary parietal cells into the secondary parietal cell layer that later converts into the tapetum. Similarly, *mil2* mutants produce an excessive number of gametes but do not develop a tapetum (Hong et al., 2012a). Both *msp1* and *mil2* genes promote periclinal cell division of the endothecium (En) and sporocytes (Sps) of neighboring somatic cells by repressing the differentiation and proliferation of AR cells. Furthermore, the gene *TDR INTERACTING PROTEIN2* (*TIP2*) encodes for a basic helix–loop–helix (bHLH) protein that performs a key role in switching the meristemoid transition during early anther differentiation process and that positively regulates meiosis and microspore release from the tetrad. Cytological studies demonstrated that *tip2* mutants have an undifferentiated endothecium, tapetum, and middle layer, which on later stages resulted in an impaired tapetal programmed cell death (PCD) and complete male sterility (Fu et al., 2014; Ko et al., 2014). Finally, the gene *TDR INTERACTING PROTEIN3* (*TIP3*) encodes for a PHD-Finger TF that is expressed during early anther development. *tip3* mutants are characterized by defective anther cuticle and pollen wall formation, an abnormal ubisch body morphology, and a delayed degradation of the tapetum (Yang et al., 2019b).

### Tapetum Differentiation and Meiotic Phase

The tapetum (T) is formed through periclinal cell division of somatic cells and corresponds to the innermost of four characteristic anther layers, which also include the epidermis (Ep), the endothecium (En), and the middle layer (ML). The tapetum surrounds the growing microspores in the anther locule and plays a key role in male gametogenesis by providing nutrients and other necessary ingredients that ensure proper pollen and anther development. Thus, any defect during the differentiation process of the tapetum results in male sterility (Ariizumi and Toriyama, 2011). The differentiation of the tapetum and meiosis occur simultaneously in rice. A number of studies have previously specified the role played by different genes, including transcription factors (TFs), during tapetum differentiation. The rice *undeveloped tapetum1* (*UDT1*) gene encodes for a bHLH TF that is involved in triggering the tapetum differentiation by terminating the periclinal cell division of somatic cells. *udt1* mutants exhibited an undeveloped and vacuolated tapetum and did not allow for the development of meiocytes into microspores (Jung et al., 2005). Similarly, rice mutants of *TDF1* (*OsTDF1*), which is an R2R3 MYB TF, displayed a defective and vacuolated tapetum, suggesting that this gene also plays a crucial role in tapetum development and male fertility (Cai et al., 2015). Furthermore, *GAMYB* encodes a transcription factor associated with the Gibberellin (GA) pathway and regulates anther development. Mutations in *GAMYB* are known to cause an expanded and undifferentiated tapetum and lead to an abnormal meiosis (Aya et al., 2009; Liu et al., 2010). Additionally, two bHLH transcription factors, *TAPETUM DEGRADATION RETARDATION* (*TDR*) and *ETERNAL TAPETUM1* (*EAT1*), are also essential for tapetum development (Li et al., 2006; Niu N. et al., 2013). Finally, the rice *MYB80* (*OsMYB80*) gene regulates anther development by targeting multiple pollen development pathways, as *myb80* mutants displayed premature PCD, an absence of Ubisch bodies, and pollen exine that resulted in no pollen production (Pan et al., 2020).

Meiosis is a crucial step for the division of PMCs, which is necessary to ensure equal chromosomal segregation to daughter cells. While the meiotic process can be crucial to determine male fertility, its role in anther and pollen wall development largely remains undetermined. Recently, a few genetic and cytological studies have evaluated the role of meiosis-related genes on pollen development. The rice gene *DEFECTIVE TAPETUM AND MEIOCYTES 1* (*DTM1*) encodes an ER membrane-localized protein and is crucial for the differentiation of the tapetum and meiotic progression during early anther development. The disruption of *DTM1* arrests the meiotic process during prophase 1 and delays ER degradation in the tapetum, which eventually causes complete male sterility (Yi et al., 2012). Furthermore, mutations in a meiosis-specific gene, *FINGL1*, cause an abnormal meiosis and lead to severe defects in the anther and pollen walls due to an excessive deposition of sporopollenin and anther cutin (Zhang et al., 2017). Finally, the gene *OsMFS1* encodes for a meiotic coiled-coil protein that is essential for the formation of double-strand breaks, which plays an indispensable role in meiosis and anther development. Accordingly, *mfs1* mutants produce abortive pollen with reduced sporopollenin deposition, an impaired tectum, and columella formation (abnormal exine) (Lu et al., 2020).

### Pollen Wall and Mature Pollen Formation Phase

#### Callose Biosynthesis and Degradation

The temporary callose layer that surrounds the newly formed microspores is synthesized by a callose synthase and facilitates microspore release from tetrads after being degraded by *b*-1,3-glucanases. Its biosynthesis, deposition, and timely degradation are important for male gametophyte development, and any abnormality during these processes can lead to male sterility. Several genes associated with callose biosynthesis have been identified in rice. The rice gene *Glucan Synthase-Like 5* (*OsGSL5*) is essential for callose biosynthesis because its mutants failed to produce a normal callose, and thus, microspores were collapsed with a defective exine patterning (Shi et al., 2015c). The recently described lectin receptor-like kinase *OsLecRK5* phosphorylates *OsUGP1* and is reportedly required for callose biosynthesis, as its mutants are male sterile and show a defective callose deposition around the microspores (Wang B. et al., 2020). Furthermore, the premature degradation of the callose layer in *dcm1* mutants caused an abnormal meiotic cytokinesis and exine formation, indicating an essential role of *OsDCM1* for callose pathway (Zhang C. et al., 2018). Recently, the *defective pollen wall3* (*dpw3*) gene was also associated with an abnormal callose biosynthesis and pollen wall formation (Mondol et al., 2020). In addition, the timely degradation of callose is necessary for the release of microspores and the formation of the pollen wall. The gene *OsG1* encodes a beta-1,3-glucanase responsible for callose degradation during late meiotic stages. Mutations in this gene cause a delayed degradation of callose and result in the production of degenerated pollen (Wan et al., 2011). Most recently, the *Defective Microspore Development 1* (*OsDMD1*) has been proven to be essential for callose degradation and pollen wall development. The *OsDMD1* directly targets *Tapetum Degeneration Retardation* (*TDR*), a key basic helix–loop–helix TF, and mediates the pollen wall formation, as the *dmd1* mutants result in delayed callose degradation and abnormally developed pollen exine and endexine, thus determines rice male sterility (Ren et al., 2020).

#### Primexine Deposition and Plasma Membrane Undulation

Deposition of primexine and plasma membrane undulation are two crucial steps to ensure the formation of the pollen wall. The callose layer encloses the microspores for protection and serves as mold for primexine patterning. By the end of meiosis, callase-mediated callose degeneration marks the starting point for the formation of primexine, an elaborate template for the deposition of sporopllenin and tryphine. The gene *OsDEX1* encodes a Ca^+^ membrane binding protein that is involved in primexine deposition and plasma membrane (PM) undulation. Interestingly, *osdex1* mutants failed to produce primexine deposition, PM undulation, and a normal exine (Yu et al., 2016). Moreover, the *Defective Pollen Wall3* (*DPW3*) gene is an ortholog of the *Arabidopsis NERD1* gene that preserves its function. *dpw3* mutants exhibit a defective formation of the pollen wall, an impaired primexine deposition, an abnormal plasma membrane undulation, and a defective callose deposition, all of which result in the accumulation of sporopollenin in the anther locule instead of the pollen surface, leading to pollen abortion (Mondol et al., 2020).

#### Lipid Metabolism

Lipid metabolism plays an important role in coordinating the development of reproductive and sporophytic tissues and the regulation of male gametogenesis. The pollen wall is constituted by lipids and their derivatives, including waxes, fatty acids, phospholipids, and many other important derived components. Exine is a complex, multilayered and biochemically encapsulated structure with a range of diverse functions during pollen development, and its biosynthesis involves a series of genes (Ariizumi and Toriyama, 2011). Among all of the cloned genes involved in lipid metabolism, most are localized in the endoplasmic reticulum (ER), although the rice genes *defective pollen wall* (*OsDPW*) and *Digalactosyldiacylglycerol Synthase* (*OsDGD2β*) are instead localized in the plastid, and the recently described *defective pollen wall2* (*OsDPW2*) is localized in the cytoplasm (Shi et al., 2011; Xu D. et al., 2017). The *de novo* synthesis of fatty acids occurs in plastids, which are then processed in the ER for the precursor synthesis of anther and pollen walls (Figure 1). Nevertheless, a fraction of lipid metabolism also occurs in the cytoplasm, and lipid transport proteins are largely localized in both the cytoplasm and the plasma membrane. The metabolism of lipids in the tapetum plays a significant role in the processing of precursors generated in plastids through a series of consecutive modifications that ensure the development of final polymerized forms of the anther cuticle and pollen wall.

##### Genes involved in lipid metabolism

Lipid metabolism is involved in the biosynthesis of sporopllenin, tryphine, anther wax, and cutin during the developments of the anther (Figure 1). Several genes are essential for sporopollenin biosynthesis, including those encoding fatty acid reductase (*OsDPW*), fatty acyl-CoA synthetase (*OsACOS12*), long-chain fatty acid ω-hydroxylase (*OsCYP704B*), cytochrome P450 fatty acid hydroxylase (*CYP703A*), glucose-methanol-choline (GMC) oxidoreductase (*OsNP1*, *OsHTH1*), and GDSL lipase (*OsRMS2*) (Shi et al., 2015a; Wan et al., 2020). Any disruption in these genes has the potential to affect their biochemical properties and leads to male sterility. *OsDPW*, *OsCYP704B2*, and *OsCYP703A3* are essential for the formation of pollen exine, while their mutants are characterized by a defective anther cuticle and pollen wall and induce the formation of defective ubisch bodies. Specifically, *OsDPW* catalyzes the reduction in fatty acy-ACP to fatty alcohols, *CYP703A3* acts as catalyst for medium-chain saturated fatty acids to the conversion of monohydroxylated fatty acids, and *CYP704B2* is an important component of sporopollenin biosynthesis through the ω-hydroxylation of C16 and C18 fatty acids (Li et al., 2010; Shi et al., 2011; Yang et al., 2014, 2018). Furthermore, loss-of-function mutations affecting *OsACOS12*, an acyl-CoA synthetase gene, result in a defective anther cuticle and affect sexine development (Li Y. et al., 2016). Additionally, the *OsNP1* and *OsHTH1* genes are essential GMC oxidoreductases that are responsible for the development of the anther cuticle and the pollen wall by participating in the oxidative pathway of short chain (C16/C18) ω-hydroxy fatty acids (Liu K. et al., 2017; Xu Y. et al., 2017). The recently reported *rice male sterile2* (*OsRMS2*) gene also seems to be involved in tapetal PCD and the formation of the anther cuticle (Zhao et al., 2020). Moreover, cytoplasmic localized *defective pollen wall2* (*OsDPW2*) encodes a BAHD acyltransferase that is important for the synthesis of polymerized protective layers of the anther and pollen walls (Xu D. et al., 2017). The *glycerol-3-phosphate acyltransferase 3* (*OsGPAT3*) has also been reported for its critical role in determining anther cuticle and the patterning of the pollen exine (Men et al., 2017; Sun et al., 2019). The *Strictosidine Synthase-Like 2* (*OsSTRL2*) gene is an important component of secondary metabolism that encodes atypical trictosidine synthases and plays an indispensable role in the maturation of the anther cuticle and the formation of the pollen exine by participating in the phenylpropanoid pathway (Zou et al., 2017a). The *Polyketide Synthases* (*OsPKS1/2*) and *TETRAKETIDE α-PYRONE REDUCTASES* (*OsTKPR1/2*) are essential for condensation and reduction in the sporopollenin metabolon to ensure the formation of the pollen exine (Zou et al., 2017b, 2018; Xu et al., 2019).

The rice gene *WAX DEFICIENT ANTHER1* (*OsWDA1*) is expressed at the epidermis and is known for its role in the decarboxylation of alkanes during the biosynthesis of anther wax. Mutations in *WDA1* cause abnormal anther epicuticular wax deposition and a defective formation of the pollen exine (Jung et al., 2006), and previous functional analysis have determined the gene has an important role on the biosynthesis pathway of very long chain fatty acids (VLCFAs) (Jung et al., 2006). Similarly, the rice gene *ECERIFERUM1* (*OsCER1*) is also an important component of the VLCFA biosynthesis pathway. Moreover, the knockdown and overexpression of *OsCER1* in plants produce a differential accumulation of VLCFAs (C25 and C28), while its downregulation causes male sterility characterized by a delayed tapetal PCD and an abnormal development of tapetal plastids (Ni et al., 2018). Additionally, the *degenerated panicle and partial sterility 1* (*OsDPS1*) gene encodes for a cystathionine b-synthase domain containing protein that has been recently associated with the development of both anther and panicle. Specifically, *OsDPS1* mutants displayed a plain anther epicuticular surface, which suggests that the gene has a potential role in the biosynthesis of the anther cutin, even though the specific pathway through which this occurs remains elusive (Zafar S.A. et al., 2020). Finally, the rice gene *Digalactosyldiacylglycerol Synthase* (*OsDGD2β*) is required for the synthesis of an important lipid digalactosyldiacylglycerol (DGDG), a major constituent of the matrix of thylakoid membranes in the endothecium of chloroplasts. Loss-of-function mutations affecting *OsDGD2β* cause complete male sterility due to a defective synthesis of DGDG that delays the tapetal PCD (Basnet et al., 2019), which suggests that this gene affects the formation of subcellular organelles in rice anthers.

##### The genetic network of lipid metabolism

In the tapetum, the *de novo* synthesis of fatty acids (C12:0, C16:0, C18: X) starts in the plastids with the reduction in fatty acids to fatty alcohols mediated by *OsDPW* (Ariizumi and Toriyama, 2011; Shi et al., 2015a; Zhang et al., 2016) (Figure 1). During this process, ACOS-like proteins (*OsACOS12*) convert C12 fatty acids into their corresponding coenzyme A, and fatty acid synthase (FAS) converts them to C16 and C18 fatty acids. These derived fatty acyl coenzyme As are then transported to the ER where they serve as the starting substrate for the synthesis of sporopollenin and tryphine. In the ER, thioesterases catalyze the conversion of fatty acyl Co-As into C16 and C18:X. Furthermore, these fatty acids undergo hydroxylation catalyzed by *OsCYP703A3* to produce 7-OH-C12:0 and ω-hydroxylation by *OsCYP704B2* to produce ω-OH-C16:0 and ω-OH-C18:X. Subsequently, the hydroxylated fatty acids are further catalyzed by the fatty acyl-CoA synthase 12 (*OsACOS12*), condensed by the *polyketide synthases* (*OsPKS1/2*), and finally reduced by the *TETRAKETIDE α-PYRONE* REDUCTASES (*OsTKPR1*) to yield esters for the biosynthesis of sporopollenins and cutin precursors (Zhang et al., 2016). Alongside this process, *OsDPW2* catalyzes the ω-OH fatty acids in the cytoplasm to produce spropollenin and cutin precursors (Xu D. et al., 2017).

The biosynthesis of sporopollenin, anther wax, and cutin share many common steps, including the *de novo* synthesis of fatty acids in plastids, acyl editing, and hydroxylation in the ER (Shi et al., 2015a). In the case of cutin biosynthesis, oxidoreductases such as *OsNP1* and *OsHTH1* oxidize ω-hydroxyl fatty acids to C16:0 diacids and C18:X diacids. Furthermore, *OsGPAT3* uses hydroxyl diacids as the starting substrate for esterification in order to produce cutin monomers. The biosynthesis of anther cuticle wax follows the VLCFA synthesis pathway. Specifically, VLCFA synthesis occurs in two separate steps. First, the FA synthases (FAS) catalyze the plastid-derived fatty acids (C16/18), and after this, fatty acyl CoA-elongase catalyzes to form VLFAs (Beaudoin et al., 2009). A fractional part of the plastid-derived C16 and C18 fatty acids remain inside the plastids as an integral part of the thylakoid membrane (Basnet et al., 2019), while the remaining are transported to the ER to synthesize VLCFAs where they follow two distinct pathways for the synthesis of cuticle wax. Further synthesis of wax from VLCFAs in the ER follows alkane- (decarbonylation) and alcohol- (acyl reduction) forming pathways. The alkane-forming pathway is catalyzed by both *OsWDA1* and *OsCER1* to produce VLC ketones for the synthesis cuticle wax through a series of successive modifications. In contrast, the alcohol-forming pathway synthesizes anther wax in a process that is probably mediated by *OsDPS1*. However, the alcohol-forming pathway of wax biosynthesis requires further investigation in rice.

##### Transportation of lipid precursors

Once the biosynthesis of sporopollenin, tryphine, wax, and cutin monomer precursors is completed in the tapetal ER, the precursor transporters rapidly transfer these lipids onto the surface of the pollen and the anther epidermis for the formation of the pollen wall and the anther cuticle (Shi et al., 2015a). In rice, this transportation is mediated by lipid transfer proteins (LTPs) and ABCG cassette transporters. The gene *OsC6*, rice LTP, is responsible for the transportation of lipidic precursors for pollen exine and anther cuticle formation. The knockdown mutants of *OsC6* produce abnormal ubisch bodies and pollen exine and exhibit a reduced fertility (Zhang H. et al., 2010). Moreover, the rice ABC transporter G3 *OsABCG3* transports sporopollenin and tryphine to the pollen. Loss-of-function *OsABCG3* mutants produce no exine and have defective nexine and intine (Chang et al., 2018; Luo et al., 2019). Additionally, the gene *OsABCG15* has been associated with the transportation of sporopollenin and cutin, and its mutants display a defective anther cuticle lacking ubisch bodies and exine (Niu B.X. et al., 2013; Wu et al., 2014). Finally, the *OsABCG26* gene is responsible for the specific transportation of wax and cutin to ensure anther cuticle maturity (Chang et al., 2016b). However, it is still unclear how they transport different precursors from the tapetum to the pollen and anther walls.

#### Intine Formation

Intine formation has been generally associated with pollen, but the exact mechanism currently remains unclear. A defective intine formation usually leads to the formation of an abnormal pollen aperture, resulting in either a poor or absent pollen germination, low seed setting, or complete male sterility. The rice *GLYCOSYLTRANSFERASE1* (*OsGT1*) and *COLLAPSED ABNORMAL POLLEN1* (*CAP1*) genes were reportedly involved in intine formation because their mutants did not produce normal intine (Moon et al., 2013; Ueda et al., 2013). Furthermore, the gene *OsUAM3*, a member of the UDP-arabinopyranose mutases (UAMs), is essential for the biosynthesis of arabinan side chains, of which pectin is an essential constituent of the intine. *OsUAM3* knockdown plants exhibited an abnormal cell wall, especially intine, because of a reduction in the number of pectin arabinan side chains and an irregular exine patterning (Sumiyoshi et al., 2015), suggesting that the gene is involved in the metabolism of polysaccharides. Another mitochondrial localized protein, *OsGCD1*, is required for intine formation since mutants for this gene produced defective intine and pollen aperture (Huang et al., 2018, p. 19). Moreover, *OsmiR528*, a conserved microRNA (miRNA) in plants, targets *OsUCL23* (a member of the phytocyanin family) and impairs the formation of pollen intine through the regulation of flavonoid metabolism. Importantly, the knockout mutants of *OsmiR528* failed to produce normal intine and fertile pollen (Zhang C. et al., 2020). Finally, another member of the phytocyanin family, *OsUCL8*, is negatively regulated by *miR408* during the formation of intine. Loss-of-function *OsUCL8* mutants produced defective intine due to a poor production of vitamin B1 and failed to develop a pollen tube during the pollen germination stage (Zhang F. et al., 2018).

#### Polysaccharide Metabolism

Starch is the major form of stored food that facilitates pollen germination by supplying energy and constituting the carbon skeleton necessary for the formation of the pollen tube and subsequent pollen germination. The starch granules start accumulating in pollen grains at late stage 11 and extend throughout stage 12 to attain maturity (Zhang et al., 2011). A defective or insufficient starch synthesis during the pollen maturation phase affects pollen germination and causes male sterility (Wu et al., 2016). Only a few genes controlling the synthesis of starch have been characterized in rice. The rice gene *hexokinase5 OsHXK5* is essential for hexokinase activity during the pollen maturation phase, and *hxk5* mutants display an upregulation of *HXK5* at this stage, which impairs starch biosynthesis and, consequently, pollen tube formation, resulting in complete male sterility (Lee et al., 2020). Similarly, mutations in the plastidic phosphoglucomutase *OspPGM* and the ADP-glucose pyrophosphorylase *OsAGPL4* genes lead to an upregulation of both genes during pollen maturation, which also affects starch biosynthesis and causes male sterility, as the pollen fails to germinate (Lee et al., 2016). These observations suggest that the manipulation of genes involved in the metabolism and biosynthesis of starch might be an ideal target to develop male sterile lines for the production of hybrid rice varieties.

### Anther Dehiscence and Pollen Germination Phase

#### Anther Dehiscence

The formation of mature pollen, along with the release of mature pollen by dehiscence, are crucial steps to ensure sexual reproduction in flowering plants, in coordination with flower opening, stamen elongation, and stomium burst. The homeostasis of phyto-hormones, including jasmonic acids (JAs) and auxins, have a significant role in anther dehiscence. Specifically, the genes *OsOPR7* and *OsJAR1* are required for anther dehiscence by mediating the biosynthesis of jasmonic acid. Mutants of both genes display defective anther dehiscence that is characterized by an impaired swelling and withering of lodicules (Tani et al., 2008; Xiao et al., 2014). Similarly, the downregulation of auxin levels during mitosis adversely affects dehiscence. The rice gene *FT-INTERACTING PROTEIN 7*(*OsFTIP7*) indirectly suppresses the expression of a prominent auxin biosynthetic gene, *OsYUCCA4*, during the later stages of anther development. The negative regulation of *OsYUCCA4* by *OsFTIP7* impairs auxin levels, which, in turn, disrupts endothecium lignification and blocks stomium burst causing anther in-dehiscence and male sterility (Song et al., 2018).

In addition, the gene bHLH142, which encodes a bHLH transcription factor, also regulates anther dehiscence. The overexpression of bHLH142 in plants affects various metabolic pathways associated with anther development, resulting in a defective septum and stomium rupture, which leads to anther in-dehiscence. This suggests that bHLH142 has a role in anther development at a posttranscriptional level (Ranjan et al., 2017).

#### Pollen Germination

Starch must be phosphorylated to a useable form in order to be transported during pollen tube formation and germination. This whole process is mediated by a carbohydrate metabolism pathway. Accordingly, insufficient or defective starch biosynthesis, degradation, or translocation leads to male sterility. Furthermore, sugar transporters (SUTs) and sucrose phosphate synthase (SPS) are involved in the apoblastic uptake of sucrose into pollen during the development of the anther, and any abnormality may also lead to male sterility. Moreover, loss-of-function mutations in the rice genes *sugar transporter1* (*OsSUT1*) and *sucrose phosphate synthase1* (*OsSPS1*) leads to defective sucrose translocation during pollen germination and hence male sterility (Hirose et al., 2010, p. 14). The pollen of *OsmiR528* mutants have a defective intine formation that later results in germination failure, reinforcing the role of intine formation for pollen germination (Zhang C. et al., 2020).

Besides polysaccharide metabolism, pollen germination is also associated with subsequent pollen hydration and pollen tube formation. Pollen hydration is facilitated by pollen aperture that permits water intake, whereby a defective aperture leads to insufficient or inexistent pollen hydration and, consequently, to poor germination. The *DEFECTIVE IN APERTURE FORMATION1* (*OsDAF1*) is a lectin receptor-like kinase gene that is required for pollen aperture formation in which loss-of-function mutations cause poor annulus formation and male sterility. Moreover, mutations in *OsDAF1* affects the colocalization of another gene, *OsINP1*, and results in the absence of the entire aperture and thus to no germination (Zhang X. et al., 2020).

### The Mechanism of Transcriptional Regulation of GMS Genes

The transcriptional regulatory network can be divided into three pathways based on the mode of activation of target genes by the TFs. Specifically, these pathways include, first, direct activation: In this pathway, the TFs directly regulate transcription by directly binding to the promoter of target genes. For example, the *GAMYB* encoding transcription factor is essential for the Gibberellin (GA) pathway and is involved in the direct regulation of expression of the genes *OsC6*, *OsCYP703A3*, and *OsKAR* (Aya et al., 2009; Zhang H. et al., 2010). Similarly, the TFs *Undeveloped Tapetum 1* (*UDT1*) and *Persistent Tapetal Cell 1* (*PTC1*) directly bind to the promoter of a GDSL lipase-encoding gene *rice male sterile2 OsRMS2/OsGELP34* and regulate its expression (Zhao et al., 2020). The genes *TDR* and *EAT1* directly regulate the expression of *OsCP1* and *OsAP25*, respectively (Li et al., 2006; Niu B.X. et al., 2013). Second is the indirect regulation: In this pathway, one TF regulates other TF(s), which, in turn, activate the expression of downstream target gene(s). The *TDR Interacting Protein 2* (*TIP2*) and *TDR Interacting Protein 3* (*TIP3*) genes follow the pathway of indirect regulation of target genes. Moreover, *TIP2* and *TIP3* regulate the expression of the *Tapetum Degeneration Retardation* (*TDR*) gene (Fu et al., 2014; Ko et al., 2014; Yang et al., 2019b), which, in turn, regulates the expression of other target genes, including *OsC6* and *OsCYP703A3*, by directly binding to their promoter. Similarly, the gene *TGA10* interacts with both *TIP2* and *TDR* and affects the expression of *AP25* and *MTR1* genes (Chen et al., 2018). Third is the coordinated regulation: Different TFs are involved in a cascade reaction by interacting with each other and synergistically regulate the expression of common target genes. For example, three bHLH proteins, specifically *TDR INTERACTING PROTEIN 2* (*TIP2*), *Tapetum Degeneration Retardation* (*TDR*), and *ETERNAL TAPETUM 1* (*EAT1*) interact with each other to form a regulatory cascade and share common target genes, such as *AP25* and *AP37* (Fu et al., 2014; Ko et al., 2014; Niu B.X. et al., 2013).

## Molecular Regulation of Environment-Driven GMS

Numerous *loci* governing environment-driven GMS (EGMS) traits have been reported to date, although only a few of them have so far been functionally characterized. The genes encoding long non-coding RNAs (Ding et al., 2012b; Zhou et al., 2014; Fan et al., 2016), MYB transcription regulator (Zhang D. et al., 2010), RNase Z^*S1*^ (Zhou et al., 2014; Jin et al., 2019), a UDP-glucose pyrophosphorylase (Chen et al., 2007), a leucine-rich repeat receptor-like kinase (Yu et al., 2017), and microRNAs (Araki et al., 2020) have been functionally characterized for TGMS, PGMS, P/TGMS, and rPGMS traits in rice (Table 2). Moreover, a couple of genes have been recently cloned (Table 2), which encode triterpene synthase (Xue et al., 2018) and *b*-ketoacyl-CoA synthase (Chen et al., 2020) and regulate humidity-mediated genic male sterility (HGMS).

**TABLE 2:** List of characterized genes for the regulation of environment-sensitive genic male sterility (EGMS) in rice.

EGMS line (S)	EGMS gene	RAP- ID	Encoding product	EGMS type	References
Nongken 58S	*pms3*	Osl2g0545900	lncRNA (LDMAR)	PGMS	Ding et al., 2012a, b
AnnongS-1	*tms5*	Os02g0214300	RNase Z^*S1*^	TGMS	Zhou et al., 2014
Peiai64S	*p/tmsl2-l*	Osl2g0545900	sncRNA (osa-smR5864)	P/TGMS	Zhou et al., 2012
9522 (*japonica* cv)	*tins 10/tms 10L*	Os02g0283800	Leucine-rich repeat receptor-like kinase	TGMS	Yu et al., 2017
Nongken 58S	*pmsl*	Osl2g0545900	lncRNA *PM1ST*, 21 nt-phasiRNAs	PGMS	Fan et al., 2016
Hejiang 19	*ugpl*	Os09g0553200	UDP-glucose pyrophosphorylase	TGMS	Chen et al., 2007
*csa* mutant	*csa*	Os01g0274800	R2R3 MYB transcription factor	rPGMS	Zhang H. et al., 2010
*osc12* mutant	*OSC12/PTS1*	Os08g0223900	Triterpene synthase	HGMS	Xue et al., 2018
*hmsl* mutant	*hmsl*	Os03g0220100	*b*-ketoacyl-CoA synthase	HGMS	Chen et al., 2020
WuxiangS	*GATA10*	Os01g0976800	GATA transcription factor (Zinc finger family)	TGMS	Jin et al., 2019
93-11S	*OsbHLH138*	Os03g0391700	bHLH DNA-binding domain containing protein	TGMS	Wen et al., 2019
HengnongS-l	*tms9-l*	Os09g0449000	PHD finger protein	TGMS	Qi et al., 2014
Peiai64S	*OsBIM2*	Os08g0490000	BIM2 transcription factor	P/TGMS	Hu et al., 2015
Mutant	*miR2118*	Non-coding Region	lnc-RNA, 21 nt-phasiRNAs, Meiosis	rPGMS	Araki et al., 2020; Zhang Y.C. et al., 2020

### Non-coding RNAs and P/TGMS Regulation

**FIGURE 2:**
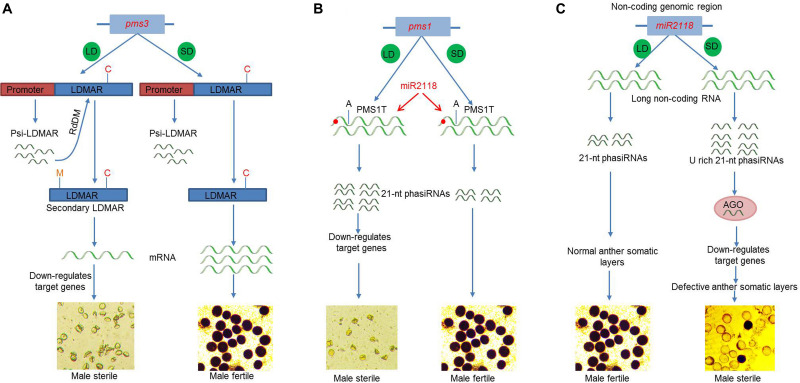
Molecular mechanism of fertility–sterility transition of environment-sensitive genic male sterility (EGMS) genes. **(A)** *pms3* encodes a non-coding RNA long-day-specific male-fertility-associated RNA (LDMAR). The promoter generated psi-LDMAR follows RdDM pathway and increases DNA methylation of single-nucleotide polymorphism (SNP) carrying LDMAR resulting in secondary LDMAR structure, reduced transcript production, and male sterility under long-day (LD) conditions. While under short-day (SD) conditions, SNP does not affect transcript production and produces fertile pollen. **(B)** *pms1* encodes a long non-coding RNA PMS1T. An SNP in the promoter of PMS1T affects the binding of *miR2118* and produces higher level of 21-nt phased small-interfering RNAs (phasiRNAs), which then downregulates the target genes and cause male sterility under long-day (LD) condition. **(C)** *miR2118* produces excess of U-rich 21-nt phasiRNAs, which, in coordination with AGO proteins, downregulates target genes and causes male sterility under SD conditions. Under LD conditions, fertility is restored. C, SNP; M, methylation; LD, long day; SG, short day; HT, high temperature; LT, low temperature; RdDM, RNA-directed DNA methylation; AGO, argonaute.

Nongken 58S was the first reported photoperiod-sensitive male sterile (PGMS) line in rice, which was caused by a G-to-C single-nucleotide polymorphism (SNP) point mutation that alters the secondary structure of a lncRNA, termed long-day-specific male-fertility-associated RNA (LDMAR) (Ding et al., 2012a). Transcriptome and deep sequencing analysis of Nongken 58S detected three small RNAs within LDMAR (Ding et al., 2012a). However, a follow-up study found that the DNA methylation patterns observed in the promoter region of LDMAR is most likely directed by a 21-nt small RNA (Psi-LDMAR), which led to reduced transcript production and thus to male sterility under long-day conditions (Ding et al., 2012b) (Figure 2A). Later, the *PMS3* locus was isolated in the TGMS line Peiai64S (which was bred by crossing NK85S and Peiai64) and named *P/TMS12-1* (Zhou et al., 2012). *PMS3* causes TGMS and PGMS in the Peiai64S and NK85S lines, respectively. The P/TGMS (Peiai64S) lines exhibit a point mutation in a 21-nt small RNA (osa-smR5864m), which may lead to loss-of-function and subsequent failure to target different genes in the *indica* and *japonica* backgrounds, and produces PGMS and TGMS, respectively (Zhou et al., 2012). Accordingly, these studies support the involvement of DNA methylation or RNA-dependent DNA methylation (RdDM) in the regulation of PTGMS.

The *pms1* locus, a 21-PHAS gene, encodes a long non-coding RNA (lncRNA) PMS1T in the PGMS rice line Nongken 58S that is specifically expressed in young panicles. Upon production, this lncRNA undergoes cleavage after being targeted by miR2118 and produces an abundance of 21-nt phased small-interfering RNAs (phasiRNAs) under long-day conditions (Figure 2B). Furthermore, a single point mutation (G to A) in the lncRNA close to the *miR2118* recognition site results in fertility–sterility transition most likely by the differential accumulation of phasiRNAs (Fan et al., 2016). Under short-day conditions, this mutation does not affect phasiRNA production and instead produces a fertile phenotype that is similar to the fertile line Nongken 58N (Fan et al., 2016).

### MicroRNAs and PGMS Regulation

Increasing evidence suggests that the previously regarded non-coding DNA (dark matter DNA) in the genome, no longer seems non-functional. For example, the microRNA2118 (*miR2118*) is conserved in plants and involved in the production of phasiRNA. Knockout *miR2118* mutants display severe male and female sterility problems in rice with marked developmental and morphological abnormalities in the somatic cell layers of the anther wall, especially under short-day conditions. In contrast, the severity of these phenotypes is lower, and plants are fertile under long-day conditions (Figure 2C). The *miR2118* mutants show an enrichment of U rich 21-nt phasiRNAs in anthers, suggesting that the loss of *miR2118* function impairs the production of 21-nt phasiRNAs and thus causes sterility. Furthermore, proteome analysis proposed that the argonaute proteins *OsAGO1b*/*OsAGO1d* may be involved in an *miR2118*-dependent 21-nt phasiRNAs biogenesis (Araki et al., 2020). These results suggest that *miR2118* presents a novel mechanism of reproductive development as an environmental response because fertility is partially dependent on the photoperiod but is independent of other photoperiodic pathways.

### RNase Z and TGMS Regulation

**FIGURE 3:**
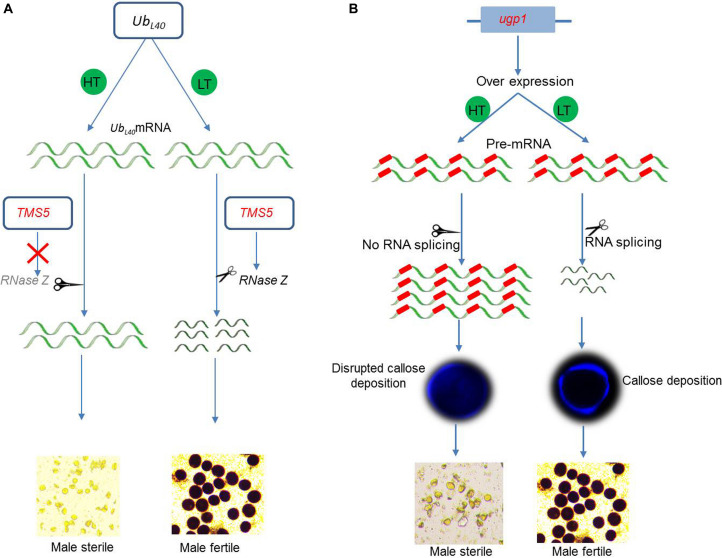
Molecular control of fertility–sterility transition of environment-sensitive genic male sterility (EGMS) genes. **(A)** *TMS5* in TGMS lines encodes RNase Z^*S1*^, which cleaves Ub*_*L40*_* messenger RNA (mRNA). The mutated tms5 does not encode RNase Z^*S1*^ and is unable to cleave Ub*_*L40*_* mRNA. Higher accumulation of unprocessed *Ub*_*L40*_ mRNA causes male sterility under high temperature (HT). Under low temperature, mutated *tms5* encodes RNase Z^*S1*^ and cleaves *Ub*_*L40*_ mRNA to produce fertile pollen. **(B)** Overexpression plants of *UPD-glucose pyrophosphorylase1* (*Ugp1*) produce abundance of unprocessed aberrant mRNA, which causes male sterility at high temperature (HT). Under low temperature (LT), the mRNA undergoes proper splicing and produces fertile pollen.

Thermosensitive genic male-sterile (TGMS) lines constitute a very important source for hybrid rice breeding. *TMS5* is a major sterility–fertility transition gene that was well investigated in a number of studies focusing on fertility transition under different temperature conditions. *TMS5* encodes a conserved RNase Z protein (RNase Z^*S1*^), which regulates the levels of accumulation of the ubiquitin-60S ribosomal protein L40 (*Ub*_*L40*_) messenger RNA (mRNA) (Figure 3A). The RNase Z^*S1*^ cleaves the ubiquitin fusion ribosomal protein L40 (*Ub*_*L40*_) mRNAs to maintain fertility. The *tms5* mutants are unable to produce RNase Z^*S1*^, which leads to an overaccumulation of unprocessed *Ub*_*L40*_ mRNAs under high temperature conditions, and results in the production of defective pollen and male sterility (Zhou et al., 2014).

The sterility–fertility transition of TGMS line (Zheda13S) derived from Peiai64S and carrying the *tms5* gene was investigated via RNA-seq analysis, which detected differentially expressed genes (DEGs) related to different cellular functions, such as protein folding, protein binding, transcription regulation, transcription factor activity, and metabolism. Furthermore, topological analysis detected *Ub*_*L40s*_ with decreased expression, DNA-directed RNA polymerase subunit, kinases, and heat shock proteins (HSPs), all of which can physically interact with each other. In summary, the sterility of Zheda13S under restrictive (high) temperature is linked to DNA-directed RNA polymerase subunits, kinases, HSPs, and other DEGs, along with a higher accumulation of *Ub*_*L40s*_ mRNA (Li et al., 2020). It can thus be concluded that there exists a number of other factors that collectively control the expression of the *tms5* gene.

### Leucine-Rich Repeats Receptor-Like Kinase and TGMS Regulation

Leucine-rich repeats receptor-like kinases are widely associated with abiotic stress responses in plants, even though their contribution to male sterility has not yet been properly studied. The rice gene *Thermosensitive Genic Male Sterile 10* (*TMS10*) and its homolog *TMS10-Like (TMS10L)* encode a leucine-rich repeats receptor-like kinase (LRR-RLK) and perform redundant functions in controlling tapetal development. Mutants of these genes produce sterile phenotypes at high temperatures and fertile phenotypes at low temperature (Yu et al., 2017). Moreover, the double mutants of *tms10* and *tms10l* exhibit complete male sterility at both high and low temperatures. These results suggest that *TMS10* is an excellent genetic resource for the production of hybrid rice varieties.

### Biochemical Regulation of HGMS

The pollen coat is the outermost layer of pollen grains, protecting the released pollen from desiccation and other environmental hazards in order to ensure a successful pollination (Pacini and Hesse, 2005). However, pollen coat composition and function is sometimes compromised by environmental fluctuations. *OsOSC12*/*OsPTS1* encodes a *triterpene synthase*, and defective *OsOSC12* mutants exhibit a reduced level of C16 and C18 fatty acids in the pollen coat and produce conditional male sterility under low relative humidity levels (Xue et al., 2018). Gas chromatography–mass spectrometry (GC-MS) analysis of pollen coat extracts revealed significantly decreased levels of palmitic (C16) and linolenic (C18) acids, despite no changes were observed in the levels of stearic acid (C18). The differential presence of these three important fatty acids caused pollen desiccation (Xue et al., 2018). Similarly, the rice gene *HUMIDITY SENSITIVE GENIC MALE STERILITY 1* (*OsHMS1*) encodes a *b*-ketoacyl-CoA synthase and regulates the biosynthesis of VLCFAs. *OsHMS1* mutants produce male sterility in consensus with decreased VLCFAs levels (C26, C28) under low humidity conditions (Chen et al., 2020). Finally, gas chromatography–mass spectrometry detected metabolic dysfunctions and pollen desiccation due to deficiency of VLCFAs, which, as mentioned above, are a major constituent of the pollen wall (Chen et al., 2020). These genes seemingly regulate a very new type of environment-sensitive GMS known as humidity-sensitive GMS (HGMS). Although these genes are involved in lipid metabolism, their expression is vulnerable to environmental changes. These genes could be the breeding source in the regions with high humidity but may not be the subject of hybrid breeding for wider application.

### Transcription Factors Regulating P/TGMS

There are number of transcription factors mediating the regulatory pathways of target genes (Table 2). The transcription factor *GATA10*, which was identified from a Wuxiang S (WXS) line derived from a *tms5* mutant allele, encodes a zinc finger protein and directly activates the expression of *Ub*_*L40*_ mRNA (Jin et al., 2019). The knockout mutant plants of *GATA10* produce low *Ub*_*L40*_ expression and tend toward fertility, suggesting that the gene is involved in the modulation of *Ub*_*L40*_ for fertility conversion. *GATA10* acts as a mediator for the interaction between *Ub*_*L40s*_ and many other TF modules to regulate TGMS traits. Another nuclear localized bHLH transcription factor, *bHLH138*, was identified in a 93-11 mutant line carrying a mutation in the second exon of the *tms5* gene. This mutant line was unable to process *Ub*_*L40*_ mRNA and exhibited typical TGMS traits. The *bHLH138* forms a basic helix–loop–helix structure, binds the core region of *tms5* promoter sequences, and activates its expression via the acidic amino-acid-rich domain (Wen et al., 2019). It is thus possible to conclude that the expression of *GATA10* and *bHLH138* can channel the expression of *tms5* and the accumulation of *Ub*_*L40*_ under fertility transition conditions. Since *GATA10* and *bHLH138* regulate the expression of *tms5*, their expression can be altered to regulate *tms5* expression to guide male fertility.

The *Carbon Starved Anther* (*CSA*) belongs to the R2R3 MYB transcription family and is required for a different type of PGMS trait. Specifically, the *CSA* regulates the expression of *OsMST8*, a gene that encodes a monosaccharide transporter and is involved in the transportation of sugars from vegetative parts to the tapetum of developing anthers. The *OsMST8* is preferentially expressed in tapetal cells and sugar-transporting vascular tissues. Mutations in the *OsMST8* gene in *CSA* lines cause complete male sterility in both *japonica* and *indica* backgrounds under short day conditions, whereas fertility under long-day conditions display a reverse PGMS (rPGMS) trait (Zhang D. et al., 2010; Zhang et al., 2013).

The *tms9-1* gene causes TGMS-like traits in HengnongS-1 and is reported as a candidate locus of *PERSISTANT TAPETAL CELL 1* (*PTC1*) and *MALE STERILITY 1* (*OsMS1*) (Li et al., 2011; Qi et al., 2014). The *PTC1* encodes a PHD finger protein that is transiently expressed in the tapetum, and its loss of function causes complete male sterility in rice (Li et al., 2011). On the other hand, *MS1* also encodes PHD finger proteins and regulates tapetal PCD and the formation of the pollen wall (Yang et al., 2019a). While *tms9-1* is most likely the candidate locus for *OsMS1* in HengnongS-1, it is necessary to implement a more detailed genetic analysis in order to validate this conclusion (Qi et al., 2014).

The rice gene *ugp1* encodes a UDP-glucose pyrophosphorylase and is required for the deposition of callose layer surrounding developing pollen. The silencing of *ugp1* causes complete male sterility that is characterized by callose disruption during meiosis. Moreover, the overexpression of *ugp1* in transgenic plants also show TGMS-like traits under normal temperatures because the endogenous primary mRNA of *ugp1* does not undergo splicing, leading to an overaccumulation of unprocessed mRNA, which cosuppresses *ugp1* (Figure 3B). The sterile status of *ugp1* can be reverted to fertility at low temperature, which is mediated by the efficient splicing of an aberrant *ugp1* mRNA (Chen et al., 2007). Therefore, the cosuppression and splicing of transcripts display a novel TGMS trait.

## Epigenetic Regulation of Different Male Sterility Systems

Epigenetics deals with chromatin marks covering DNA methylation and posttranscriptional histone modifications that represent key epigenetic tools in the modulation of chromatin accessibility to replication, transcription, DNA repair, and expression of genes and transposable elements (TEs) (Joshi et al., 2016; Shriram et al., 2016). Recently, an increasing number of studies explored the genetic basis of male sterility in rice (Table 1). Genome-wide DNA methylation, histone modification, and small interference RNAs are the most prominent characteristics of the epigenetic modification landscape in rice. These modifications comprehensively determine epigenetic regulation patterns and ultimately regulate gene expression (Banerjee and Roychoudhury, 2017).

### DNA Methylation

**FIGURE 4:**
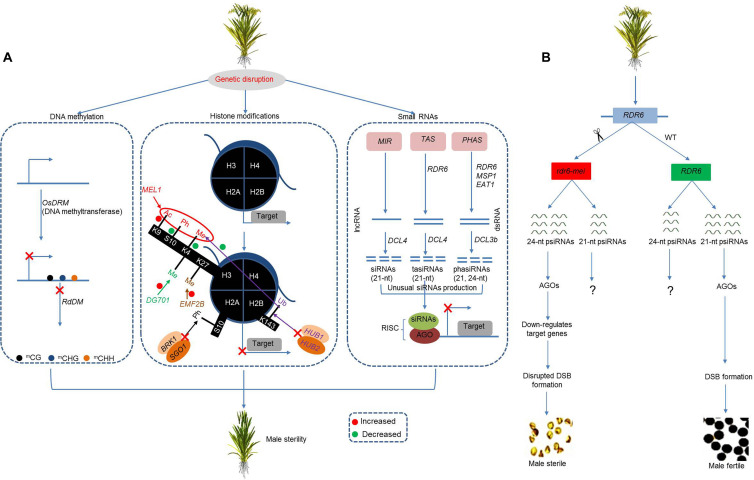
Integrative model of the epigenetic regulation of male sterility in rice. **(A)** Genetic disruption in epigenetic-related genes diverges DNA methylation pattern and causes impaired histone modification and unusual production of small-interfering RNAs (siRNAs). The divergent epigenetic pathways cause gene silencing/inability to activate target genes and cause male sterility. **(B)** The mutation in *OsRDR6* causes increased production of 24-nt psiRNAs, which, in coordination with AGO proteins, downregulates target genes, affects DSB formation, and causes male sterility. RdDM, RNA-directed DNA methylation; Ac, acetylation; Ph, phosphorylation; Me, methylation; Ub, ubiquitination; MIR, microRNA locus; AGO, Argonaute; lncRNA, long non-coding RNA; dsRNA, double-stranded RNA; RISC, RNA-induced silencing complex.

DNA methylation contributes to environmentally induce phenotypic variations by regulating gene expression through epigenetic modification (Figure 4A). DNA methylation and whole-genome sequencing analysis have been widely applied to the study of male sterility in rice. A photoperiod-thermosensitive genic male sterile (PTGMS) line (PA64) shows a higher proportion of differentially methylated regions at high temperatures and long-day conditions and thus is sterile (Chen et al., 2014). Moreover, the rice gene *BIM2* (*OsBIM2*) encodes a transcription factor *BIM2* and is involved in brassinosteroid signaling. The hypermethylation of *OsBIM2* has also been suggested to be involved in the impairment of brassinosteroid signaling and ATP production during pollen development in the PTGMS line PA64 under restrictive conditions (Hu et al., 2015).

Mutant studies have supported a regulatory role for DNA methylation in the process of male reproduction in rice. The rice gene *DOMAINS REARRANGED METHYLASE 2* (*OsDRM2*) encodes DNA methyltransferases and is required to maintain non-CG methylation (CHG and CHH). Targeted disruption of *OsDRM2* severs *de novo* methylation and produces pleiotropic developmental defects that result in complete male sterility along with vegetative growth defects. In addition, the *osdrm2* mutant shows a deficiency in the RdDM pathway and reduced levels of genome-wide DNA methylation when compared to wild-type plants and shows a higher expression in the reproductive parts by downregulating many target genes (Moritoh et al., 2012; Anderson et al., 2013).

### Histone Modifications

Histones are the major protein components of the chromatin, being responsible for its structure and functional state. Posttranslational histone modifications include ubiquitination, acetylation, phosphorylation, and methylation (Kouzarides, 2007). A variety of genomic analyses have measured the epigenetic responses on several genes associated with male reproductive development in rice (Table 3).

**TABLE 3:** List of characterized genes for epigenetic regulation of genic male sterility (GMS) in rice.

EGMS gene	RAP-ID	Encoding product	Biol. function	MS type	References
*OsDRM2*	Os03g0110800	DNA methyltransferases	DNA methylation	GMS	Moritoh et al., 2012; Anderson et al., 2013
*OsMSP1*	Os01g0917500	LRR-RLKase	phasiRNAs production	GMS	Fei et al., 2016
*OsDCL4*	Os04g0509300	RNase III-like enzyme	tasiRNA production, Meiosis	GMS	Liu et al., 2007; Zhang Y.C. et al., 2020
*EAT1*	Os04g0599300	bHLH transcription factor	Transcription of 24-PHASs	GMS	Ono et al., 2018
*OsHUB1*	Os04g0550400	E3 ubiquitin ligases	H2B monoubiquitination	GMS	Cao et al., 2015
*OsHUB2*	Osl0g0565600		Histone di-methylation		
0*sMOF*	Os04g0464966	E3 ubiquitin ligases	Histone ubiquitination	GMS	He et al., 2016
*OsMEL1*	Os03g080020	Argonaute proteins	Histone phosphorylation	GMS	Komiya et al., 2014; Liu and Nonomura, 2016
			Histone di-methylation		
			Histone acetylation		
*OsBRK1*	Os07g0508500	Ser/Thr protein kinase	H2A phosphorylation	GMS	Wang et al., 2012
*OsEMF2B*	Os09g0306800	lncRNAs	Histone methylation	GMS	Conrad et al., 2014; Johnson et al., 2018
*OsSDG701*	Os08g0180100	H3K4 methyltransferase	Histone tri-methylation	GMS	Liu Z. et al., 2017
0*sDC*Z*3b*	Osl0g0485600	Small ncRNAs	tasiRNA production	GMS	Liao et al., 2019
*OsRDR6*	Os01g0527600	RDR polymerase	tasiRNA, 24nt-siRNAs	GMS	Song et al., 2012a

Two histone H2B monoubiquitination E3 ligases, *OsHUB1* and *OsHUB2*, have been attributed a regulatory role during male reproductive development in rice (Cao et al., 2015). Specifically, loss-of-function *OsHUB1* and *OsHUB2* mutants cannot produce H2Bub1 and show a reduction in histone3 lysine-4 dimethylation (H3K4me2). Additionally, *OsHUB1* directs histone H2B monoubiquitination (H2Bub1). Genetic analysis detected a depletion of H3K4me2, some DEGs related to tapetum degradation, and binding of H3K4me2 to the promoters of these genes. These results suggested that *OsHUB1* and *OsHUB2* function as E3 ligases to regulate histone H2B monoubiquitination. Depletion of H2Bub1 affects the expression of several genes during anther development, impairs gene transcription via H3K4me2, and results in complete male sterility (Cao et al., 2015). The rice gene *MEIOTIC F-BOX* (*OsMOF*) is an important E3 ligase that recruits *completion of meiosis I* (*COM1*) and *radiation sensitive 51C* (*RAD51C*) genes, diverges the ubiquitination machinery, arrests meiotic progression, and causes epigenetic double-stranded breaks (DSB) and complete male sterility in knockout mutant plants (He et al., 2016). However, details on the underlying ubiquitination mechanism are yet to be determined.

Meiosis is an indispensable process during male reproductive development in rice and is regulated by epigenetic factors. The *MEIOSIS ARRESTED AT LEPTOTENE1* (*MEL1*) gene encodes an argonaute (AGO) protein that is specifically involved in controlling germ-cell development and meiosis in rice (Nonomura et al., 2007). *MEL1* is expressed during the premeiotic phase of cell division, irrespective of gender (Nonomura et al., 2007; Komiya et al., 2014). A study performing deep sequencing of small RNAs associated with *MEL1* has highlighted that the gene preferentially binds to a unique group of 21-nucleotide (nt) siRNAs carrying a conserved cytosine residue at the 5′-terminus that is highly expressed in rice reproductive organs. The loss-of-function *MEL1* mutants display abnormal sporophytic and germ cell development (Komiya et al., 2014), and aberrant meiotic patterns have been observed in *mel1* rice mutants during male gametophyte development (Liu and Nonomura, 2016). The transition of premeiosis to meiosis in PMCs is compromised due to an elevation in H3K9me2 and a reduction in both acetylation of lysine 9 of histone 3 (H3K9) and phosphorylation of histone 3 (H3S10) (Liu and Nonomura, 2016). These well-coordinated and comprehensive epigenetic modification events modulate *MEL1*, which preferentially binds 21-nt phasiRNAs. The meiotic chromosome reprogramming is severely affected in the anthers of *mel1* mutants, which ultimately disturbs important meiotic events, such as the presynaptic centromere association, the initiation of DNA double-strand breaks (DSBs), and the pairing of homologous chromosomal. The rice *mel1* mutants are also characterized by an abnormally developed tapetum, an unusual vacuolation of the PMCs, and a deficient chromosomal condensation during early meiosis (Nonomura et al., 2007; Komiya et al., 2014; Liu and Nonomura, 2016; Zhang Y.C. et al., 2020).

The budding uninhibited by *benzimidazole 1* (*Bub1*), a conserved Ser/Thr protein kinase, has been reported for phosphorylation of histone H2A (Kawashima et al., 2010). In rice plants, the *Bub1-RELATED KINASE1* (*BRK1*) gene encodes a Ser/Thr kinase and is essential for the phosphorylation of H2A by engaging *SHUGOSHIN1* (*SGO1*). Furthermore, the inner centromeric histone H3-S10 does not undergo phosphorylation at the diakinesis stage in *brk1* mutants, produces erroneous kinetochore–microtubule attachment, results in an unequal segregation of chromosomes, and produces male sterile phenotypes (Wang et al., 2012). *SGO1* regulates the required homeostatic tension between the homologous kinetochores specifically at metaphase I and ensures accurate chromosomal segregation at the transition between metaphase I to anaphase I (Shi et al., 2015b).

Polycomb repressive complexes 2 (PRC2) are the only known protein family that modulates chromatin structure and, consequently, regulates target gene expression through H3K27me3 in plants (Margueron and Reinberg, 2011; Schwartz and Pirrotta, 2013). A number of genes belonging to the PRC2 protein group regulate flower development and include mutations that cause defective floral progression in plants (Tariq and Paszkowski, 2004; Liu Z. et al., 2020). In the specific case of rice, the loss-of-function mutant of *EMF2B* (*OsEMF2B*) shows complete sterility through H3K27me3-mediated enrichment of the E-function genes *OsMADS1* and *OsMADS6* in anthers and modulates their reproductive function (Conrad et al., 2014). Furthermore, RNA-seq analysis identified numerous long intergenic non-coding RNAs (lincRNAs), many of which belong to the PRC2 class, suggesting that they are targeted (rather than being regulated) by PRC2 and produce abnormal reproductive development (Johnson et al., 2018). Histone3 lysine4 di/tri-mrthylases (H3K4me2/3) are distributed within the rice genome euchromatin, and any disturbance can lead to severe phenotypic defects by activating other genes (Cui et al., 2013; Liu et al., 2015). The rice gene *SET DOMAIN GROUP 701* (*OsSDG701*) encodes a H3K4-specific methyltransferase, binds chromatin to enhance H3K4me3 enrichment, promotes the expression of other florigenes (*Hd3a* and *RFT1*), and impairs the development of male gametes (Liu Z. et al., 2017).

### Phased Secondary siRNAs

Phased secondary siRNAs (phasiRNAs) play a key role in the regulation of plant reproductive development. The biogenesis of phasiRNAs is triggered by a 22-nt microRNA that causes an AGO protein-mediated silencing of the precursor locus PHAS. These process further recruits’ enzymes that are necessary for making sliced double-stranded mRNA that is further processed by Dicer-like (DCL) proteins into 21 and 24-nt siRNAs. These small RNAs form an RNA-induced silencing complex (RISC) with the AGO protein for transcriptional (TGS) or posttranscriptional gene silencing (PTGS). The 24-nt phasiRNAs are usually involved in meiotic stages of anther development, while 21-nt phasiRNAs play an important role during the premeiotic developmental stages of the anther and drive photosensitive male sterility (Xia et al., 2019).

The rice *MULTIPLE SPOROCYTES1* (*OsMSP1*) gene encodes a leucine-rich-repeat receptor kinase and plays an important role in the development of anthers. This role has been detected in a study looking at an *Osmsp1* male sterile mutant, which detected a depletion of *miR2275* that is important to trigger the production of 24-nt phasiRNAs from PHAS loci. The depletion of *miR2275* resulted in a depletion of 24-nt phasiRNAs and its precursor *PHAS* (Fei et al., 2016). Further expression analysis through RNA-seq found three distinct AGO protein encoding genes, *OsAGO1d*, *OsAGO2b*, and *OsAGO18*, suggesting a functional relationship between these genes and phasiRNA in the developmental control of male reproduction (Fei et al., 2016).

The rice *Dicer-Like4* (*OsDCL4*) gene encodes an RNase III-like enzyme that catalyzes the processing of siRNA precursors via specific biogenesis and works in association with inverted repeat transgenes and the endogenous *TRANS-ACTING siRNA3* (*TAS3*) to produce 24-nt siRNAs and phasiRNAs to regulate male reproductive development (Liu et al., 2007; Song et al., 2012a; Zhang Y.C. et al., 2020). The rice *RNA-dependent RNA polymerases6* (*OsRDR6*) gene is involved in the biogenesis of variety of small RNAs, and the gene largely controls the production and accumulation of small RNAs including tasiRNAs from the TAS locus, and 21 and 24-nt phased small RNAs from PHAS locus (Song et al., 2012b).

An allelic *OsRDR6* mutant (*rdr6-mei*) causes meiotic defects by blocking the formation of DSB. Specifically, the mutant produces decreased 21-nt RNAs and an increased proportion of 24-nt RNAs that bind to AGO protein family members and downregulate the expression of genes involved in DSB formation, which ultimately affects meiotic progression and causes no pollen type complete male sterility (Figure 4B). Furthermore, genetic analysis identified numerous differentially methylated regions, supporting a DNA methylation role for *OsRDR6*, which is independent of RdDM (Liu C. et al., 2020). Similarly, the biochemical function of *Dicer-like3b* (*OsDCL3b/OsDCL5*) is to process small RNAs (21 and 24 nucleotide) in rice panicles (Song et al., 2012a). The knockdown of *OsDCL3b* affects the biogenesis of 21- and 24-nt sRNAs, along with phased small RNAs and miRNAs. In addition, the downregulation of *OsDCL3b* hampers the transcription of genes related to panicle development (namely, *CYP704B2* and *OsPKS1*), which consequently reduces pollen fertility and seed setting rate (Liao et al., 2019).

The *ETERNAL TAPETUM1* (*EAT1*) is a bHLH transcription factor that regulates postmeiotic PCD in the anther tapetum of rice plants. *EAT1* promotes the transcription of 24-*PHAS* RNAs and also activates *DICER-LIKE5* (*DCL5*), which is known for processing double-stranded 24-*PHAS*s into 24-nt lengths. Moreover, *EAT1* can bind to the chromatin region of two transcription factors (*TIP2* and *UTD1*) and act as a key regulator of meiotic phasiRNA biogenesis in the anther tapetum, as well as other bHLH proteins to regulate anther development (Ono et al., 2018).

## Biotechnological Strategies to Develop Male Sterility and Hybrid Breeding in Rice

### Seed Production Technology for Next-Generation Hybrid Production

**FIGURE 5:**
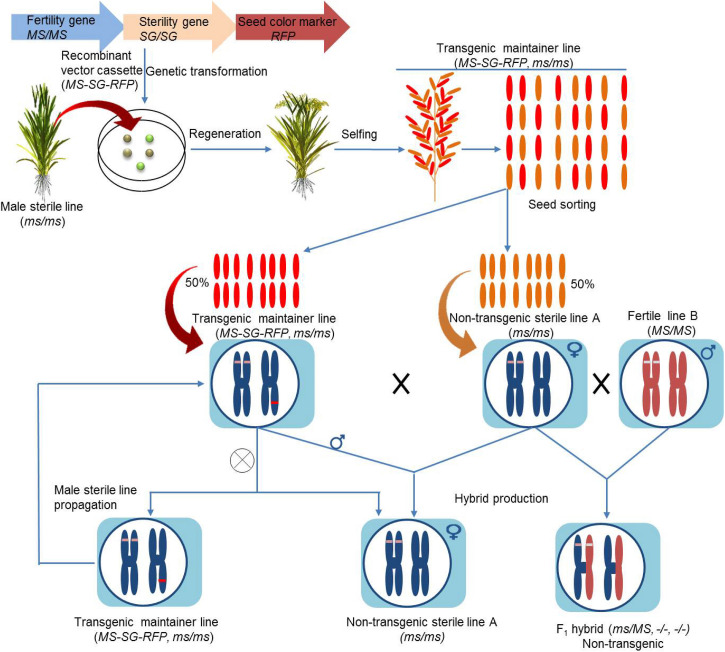
Illustration of seed production technology (SPT) using transgenic construct-driven non-transgenic hybrid strategy in rice.

The CMS and PTGMS are the major sterility systems being extensively applied for second-generation hybrid seed production. However, the utilization of CMS resources for hybrid seed production has a low efficiency, and PTGMS lines are often affected by environmental fluctuations. Moreover, these hybrid production methods are time consuming and cumbersome, which poses restrictions to their wider applications. The next generation male sterility-mediated hybrid production technology is an excellent platform to differentiate between GMS and maintainer seeds/lines based on seed production technology (SPT) methods. The SPT maintainer line is developed by combining (1) a wild-type fertility restorer gene, (2) a pollen killer gene, and (3) a marker gene to identify transgenic and non-transgenic seeds (Figure 5). The next generation hybrid production using SPT has streamlined the hybrid production process by taking advantage of the CRISPR/Cas system.

The transgenic construct-driven non-transgenic system of SPT has been devised and successfully applied in rice. The rice *No Pollen 1* (*OsNP1*) gene encodes a glucose–methanol–choline oxidoreductase and is specifically expressed in the tapetum and microspores. *OsNP1* has been used to construct a male sterility system by coupling the *OsNP1* as a fertility restoration gene with an alpha-amylase gene (*zm-aa1*) to debilitate transgenic pollen and a red fluorescence protein (DsRed) gene to identify transgenic seeds. This gene construct was transformed into the *osnp1* mutant to develop a transgenic SPT line, which resulted in 50% non-transgenic fertile pollen and 50% transgenic sterile pollen carrying the sterility gene. All of the produced pollen grains had an *np1* genotype. The non-transgenic SPT line carried a single hemizygous transgene, and its selfing produced male sterile genotype (*np1/np1*) and SPT fertile genotype (*np1/np1+SPT-TDNA-RFP*) in 1:1. The crossing of transgenic plants raised from the fertile seeds (SPT), selected based on red florescence from self-pollinated seeds of the transgenic rice line carrying the *osnp1* sterile mutant (*np1/np1*), produced male sterile plants. Around 85% of the F_1s_ of these transgenic male sterile plants crossed with fertile cultivars of different backgrounds outperformed the parental generation by 10% (Chang et al., 2016a). The male sterile mutant *osnp1* and the transgene locus can be easily transform into different genetic backgrounds and facilitate the breeding of male sterile lines (Chang et al., 2016a).

Although this SPT system is associated with many potential advantages, the transmission rate of the transgene through the pollen is equal to zero, and the red-florescence-based sorting of transgenic seeds minimizes the transmission of transgenes. Still, there exists a risk associated with transgene flow, whereby the utilization of this system can be restricted in regions with strict bioethics policies.

In order to circumvent these limitations, a novel SPT-based technique was established to develop a third generation hybrid (Song et al., 2020). The rice male fertility gene *CYP703A3* encodes a cytochrome P450 hydroxylase and is critical for pollen development. Furthermore, *OrfH79* is a well-characterized CMS gene in the Honglian CMS (HL-CMS) system in rice (Peng et al., 2010; Wang et al., 2013). The aforementioned strategy used *CYP703A3* as a fertility restorer, *ofH79* as a pollen killer, and DsRed2 as the selective marker gene. Accordingly, the clustered regularly interspaced short palindromic repeats associated endonuclease Cas9 (CRISPR/Cas9) system was used to construct a recombinant vector cassette carrying *CYP703A3*, *OrfH79*, and DsRed2 that was later transformed into separately developed complete male sterile *CYP703A3* mutants to develop a maintainer line. The selfing process of this maintainer line produced both transgenic (fertile) and non-transgenic (sterile) seeds at a 1:1 ratio. The crossing of sterile lines with other elite cultivars (male) produced hybrids with a 13% increase in yield compared to the parental lines (Song et al., 2020). Hence, the application of a CMS gene successfully created a transgene-free plant, outperforming the hybrid plant that is entirely compatible with the “zero tolerance” policy of genetically modified organisms (GMOs) production.

Seed production technology is a great step toward hybrid seed breeding, but not all of the promoters of male specific genes are able to drive the expression of sterility genes, which is a limiting factor for the widespread use of SPT. Strategies should be adopted for the selection of an effective promoter. Focusing on the importance of the use of specific promoters in the SPT-based hybrid production, several methods are being developed to select a strong promoter to drive the expression of pollen killer genes that enable the construction of a SPT maintainer line. For example, Wang M. et al. (2020) used comparative RNA-seq analysis on meiosis-related male sterility genes to select late-stage pollen-specific promoters (LSPs) and identified promoters that could drive the expression of the *ZmAA1* gene using DsRed as the marker gene. To harvest the maximum potential of SPT, the identification of promoters thus provides a valuable tool for genetic manipulation of male sterility systems for the production of hybrid rice.

### *BARNASE/barstar* and *Cysteine Protease/Cystatin* System for Dominant Male Sterility

**FIGURE 6:**
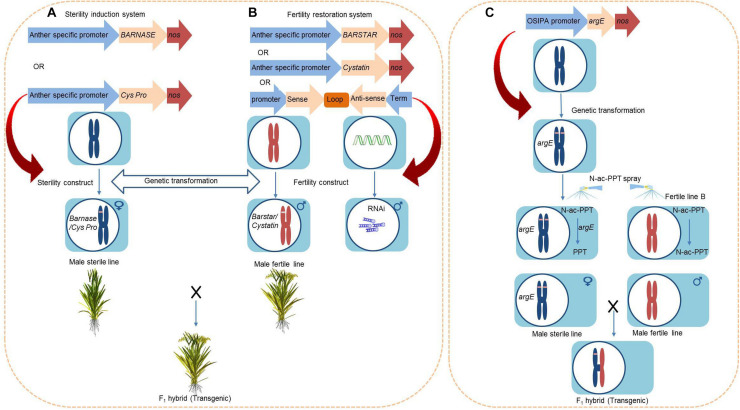
Work flow for the construction of transgenic male sterility. **(A)** *Barbase/Barstar* or *Cysteine Protease/Cystatin*. **(B)** Fertility restoration system. **(C)** Chemically induced male sterility system using *argE* gene.

The *BARNASE/barstar* system uses the *BARNASE* gene from *Bacillus amyloliquefaciens*. The gene encodes a toxic ribonuclease RNase and has been used to create dominant male sterility (DMS) (Figure 6A). This system was initially designed for tobacco by fusing the tapetum-specific promoter T29 with the *BARNASE* gene in order to create male sterility. This strategy was later extended too many crop species including rice, maize, alfalfa, and *Brassica* (Mariani et al., 1990). The ribonuclease RNase targets tapetum, degrades tapetal RNA, and triggers a premature PCD of the tapetum, resulting in male sterility. The coexpression of *BARNASE* and *barstar* reverses the expression of the *BARNASE* gene and restores fertility in the F_1_ generation (Figure 6B). The *BARBASE* system of transgenic male sterility has achieved lot of success in rice, with a total of 38 anther-specific genes being identified after screening the RiceXPro database (Akasaka et al., 2018). Linking the promoter regions of all of these genes to the *barnase* gene and expressing it in transgenic rice lines led to male sterility in lines showing expression at early anther developmental stages and exhibited strong flowering habits (Akasaka et al., 2018). The identified genes can be efficiently used to generate male sterile lines and facilitate out-breeding in rice.

The *OsC6* is a tapetum-specific gene that encodes a lipid transport protein. The fusion of *OsC6*’s promoter with the *BARNASE* gene causes complete male sterility in rice (Kumar et al., 2017). Moreover, the *Zm13* is a pollen-specific gene in maize that is expressed in the late stages of pollen development. Fusing the promoter of *Zm13* (*Zm13pro*) with the cytotoxic *BARNASE* gene and transforming into *japonica* rice background displayed stable male sterility, which indicates the stable expression of the transgene (Hague et al., 2012). Similarly, the gene *BoA9* (from *Brassica oleracea* L.) is a tapetum-specific promoter that is responsible for male fertility. The linking of *BoA9* with the *BARNASE* gene and subsequent genetic transformation into Nipponbare genetic background resulted in complete male sterility. Furthermore, the open cross-pollination of this sterile male line with fertile plants produced < 1.5% seed setting. In contrast, emasculation and manual pollination resulted in more than 86% seed setting (Abe et al., 2018).

Similarly, in the cysteine protease/cystatin system, the tapetum-specific expression of a cysteine protease impairs the development of the tapetum and causes male sterility (Figure 6A). The *Os12bglu38* gene is specifically expressed in the anthers of rice, while the *Brassica napus cysteine protease 1* (*BnCysP1*) is expressed during tapetal PCD and causes male sterility. The fusion of *Os12bglu38*’s promoter (P1) with *BnCysP1* and transformation into a cultivated rice variety resulted in complete male sterility without producing harmful effects on female fertility (Rao et al., 2018). The coexpression of *cystatine* with *cysteine protease* system (Shukla et al., 2016) or the crossing of male sterile lines with other transgenic rice lines that were developed by silencing *BnCysP1* through RNA interference (RNAi) are able to restore fertility in the F1 generation (Rao et al., 2018) (Figure 6B). Over the past decade, several genes specifically associated with anther development have been identified. The application of these genes in integration with biotechnological approaches can accelerate the production of male sterile lines and hybrid cultivation.

Moreover, the DMS can promote recurrent selection (RS), which is an excellent method to improve the quantitative traits of the population for multiple characteristics. However, the involvement of dozens of parental lines and inefficient crossing techniques has restricted its utilization. The application of RS breeding can be improved by the introduction of GMS in rice (Grenier et al., 2015). The DMS can accelerate the efficiency of RS, but the dominant GMS genes have rarely been found in rice breeding system (Yang et al., 2012; Pang et al., 2017). Hence, DMS through *BARNASE/barstar* and *cysteine protease/cystatin* system would facilitate future RS breeding strategy for the development of lines with potential resistance against multiple biotic and abiotic stresses.

### Chemically Induced Male Sterility Mediated by Anther-Specific Conversion Gene

The *Escherichia coli argE* gene deacetylates the non-toxic compound *N*-acetyl-L-phosphinothricin (*N*-ac-PPT) and converts it to a toxic herbicide compound that is harmful to the cells and known simply as phosphinothricin (PPT). The *argE* gene is expressed in tapetum cells of transgenic tobacco and results in male sterility upon treatment with *N*-ac-PPT because of the accumulation of PPT in the tapetum (Kriete et al., 1996). The promoter of the *Oryza sativa indica pollen allergen* (*OSIPA*) contains various *cis*-regulatory elements and is involved in different anther developmental stages in tobacco, *Arabidopsis*, and rice (Swapna et al., 2011). The expression of the *argE* gene under the activity of the *OSIPA* promoter produced an inducible male sterility system in rice (Figure 6C). Specifically, the transgenic rice plants underwent complete sterility when treated with *N*-ac-PPT, but this process did not affect female fertility. The restoration of male fertility does not require a fertility restorer line, as *argE* lines that were not treated with PPT are fertile and can propagate female (sterile) lines (Rao et al., 2017).

### Developing High-Throughput Male Sterile Mutant Libraries

The advancement in functional genomics has helped develop high-quality database annotations that can be subjected to next-generation sequencing in order to identify target genes and develop whole-genome-scale mutant libraries. The traditional methods of mutant library development are mainly based on the generation of random mutations via mutagens such as ethyl methanesulfonate (EMS), irradiations, T-DNA insertions, transposons, and chemical treatment. However, obtaining a stable expression of loss-of-function mutations and determining the relationship between genotype and phenotype in these traditional mutants is both time consuming and laborious. Recently, CRISPR/Cas9 has helped establish genome-wide mutant libraries (Lu et al., 2017; Meng et al., 2017). Ma et al. (2019) screened the RiceXPro^1^ database using bioinformatic techniques and microarray analysis and identified a total of 1078 genes specifically expressed in the anther. Of these, 555 showed specific expression at the trinucleate stage of anther development, while the remaining 523 genes were expressed before the trinucleate stage. Finally, 73 anther-specific genes that were highly expressed during meiosis and the uninucleate stage of microspore development were knocked out using CRISPR/Cas9 and transformed into *japonica* rice variety Zhonghua 11 (ZH11) by *Agrobacterium*-mediated transformation. A total of 15 (out of 73) mutants displayed diversified male sterile phenotypes, including partial sterility, typically abortive sterility, no-pollen sterility, and no-anther sterility (Ma et al., 2019). These observations suggest that the creation of whole-genome rice male sterile mutant libraries offers numerous possibilities for functional genomics research and the development of innovative germplasm resources for breeding hybrid rice in the future.

### Accelerating a Two-Line Breeding System Using CRISPR/Cas9

High-quality hybrid breeding to create resistance against biotic and abiotic factors has always been a preferable approach for the improvement of crop productivity. The availability or generation of a stable male sterile line is the prerequisite to produce high-quality hybrids. Over the years, EGMS lines have been broadly used for two-line systems of hybrid breeding because of (1) availability of germplasm resources and (2) reversibility of fertility via the manipulation of environmental factors (Chen and Liu, 2014; Xue et al., 2018; Zhu et al., 2020; Zhang Y. et al., 2020). On the other hand, abrupt environmental fluctuations have always posed a serious threat for their wider applications. A number of genome editing tools have been designed to manipulate the genetic code. Of these, the CRISPR/Cas9 is the most efficient tool to enable genome editing in plants (Belhaj et al., 2015; Weeks et al., 2016; Zafar K. et al., 2020).

The CRISPR/Cas9 system has been applied to create specific mutations in a thermosensitive gene *TMS5* and resulted in the development of 11 new TGMS *indica* rice lines within one growing year (Zhou et al., 2016). Recently, a very similar experiment used CRIPSR-Cas9 to edit the *TMS5* gene and generated two TGMS lines in an *indica* rice cultivar background (Barman et al., 2019). Moreover, CRISPR/Cas9 multiplex gene editing technology has assisted in pyramiding the *TMS5* gene with disease-resistant genes to generate thermosensitive disease resistance male sterile line. For example, the recessive *pi21* (Fukuoka et al., 2009) and *xa13* (Chu et al., 2006) provide resistance against rice blast and bacterial blight, respectively. The triple *tms5*, *pi21*, and *xa13* mutant displayed TGMS-like traits and also enhanced resistance against rice blast and bacterial blight (Li et al., 2019). The *CSA* is a prominent gene for the development of rPGMS-like traits exhibiting male fertility under long-day conditions and male sterility under short-day conditions in *japonica* rice. The manipulation of *CSA* by the CRISPR/Cas9 technology developed two reverse PGMS lines, namely, 9522csa and JY5Bcsa, and one rPTGMS145 line, specifically KY131csa-4 (Li Q. et al., 2016). Furthermore, the photo-thermosensitive GMS gene *P/TGMS2-1* was cloned from a widely used P/TGMS line Peiai64S. The editing of *ptgms2-1* by CRISPR/Cas9 created two new P/TGMS lines in two different *indica* backgrounds (Lan et al., 2019). Thus, CRISPR/Cas9 can efficiently be applied to manipulate EGMS genes through targeted disruption to produce hybrids with the desirable improved traits.

Multiplex genome editing using CRISPR/Cas9 has the possibility to edit multiple targets simultaneously (Zsögön et al., 2018), which could efficiently assist in pyramiding the several genes of interest along with male sterility genes to develop male sterile lines for broader benefits in rice breeding. Despite the robust benefits of the technology, the use of CRISPR/Cas9 has concerns of off-target mutations in the unintended sequences. In plants, however, such unexpected mega-alterations have not yet been observed, but the probability must be taken into account to avoid deleterious effects of the technology.

**FIGURE 7:**
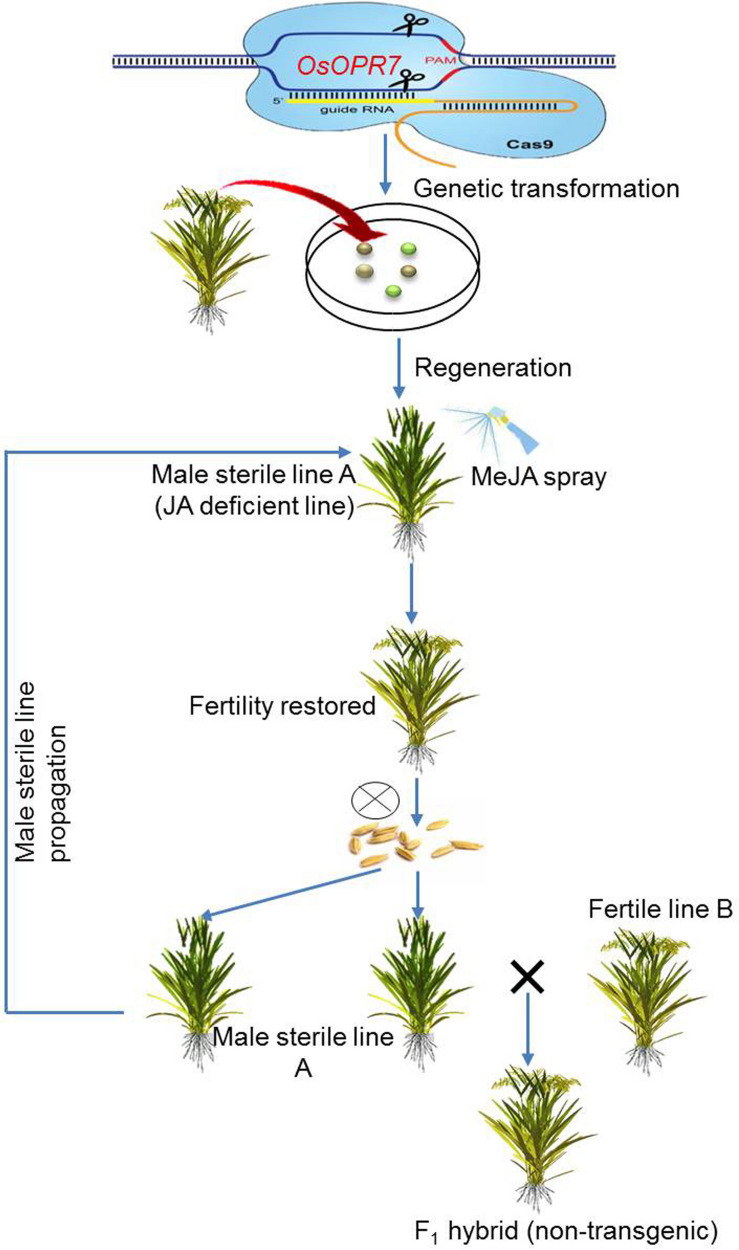
Phytohormone genic male sterility system for two-line hybrid production. Exogenous application of MeJA on jasmonic-acid-deficient male sterile mutants rescues the fertile phenotype for sterile line propagation and hybrid production. JA, jasmonic acid; MeJA, methyl jasmonate.

More recently, a strategy was proposed to create male sterile lines by disrupting the biosynthesis of jasmonic acid. Specifically, *OsOPR7* encodes a 12-oxophytodienoate reductase, which is a precursor for the biosynthesis of JA (Tani et al., 2008). *osopr7* mutants created using CRISPR/Cas9 displayed anther indehiscence and complete male sterility. The exogenous application of methyl jasmonate (MeJA) makes it possible to restore fertility and facilitates seed setting through the selfing of mutant plants (Pak et al., 2020). The seeds can then be used for the propagation of male sterile lines and hybrid production by crossing the male sterile lines with desirable fertile lines (Figure 7). Importantly, this male sterility system is insensitive to environmental effects and the application of MeJA is not hazardous to human health. Hence, this system can be widely applied to the production of hybrid rice crops in the future.

The manipulation of *OsOPR7* successfully produced phytohormone genic male sterility and then transgene-free hybrid, suggesting its possible application for other genes such as *OsFTIP7* because its auxin-deficient mutant and *osopr7* mutant have consistent anther indehiscence phenotype, whereas the majority of pollen in both of the mutants remains fertile. Both the mutants exhibit mitotic disorder, which can be reversed through exogenous application of concerned phytohormone. However, the idea may not necessarily work for other phytohormones-related genes. For example, the Gibberellins (GAs)-deficient mutant *Osatg7-1* is male sterile characterized by significantly reduced GA contents and defective microsporogenesis. The exogenous application of GAs cannot restore pollen fertility (Kurusu et al., 2017; Hanamata et al., 2020). The different behavior of different phytohormone male sterile mutants suggests that the application of technology will largely depend on the underlying genetic cause of male sterility, hence can be applied to the selected genes, which demands enhanced understandings.

### Maintaining Hybrid Vigor Through Synthetic Apomixes

**FIGURE 8:**
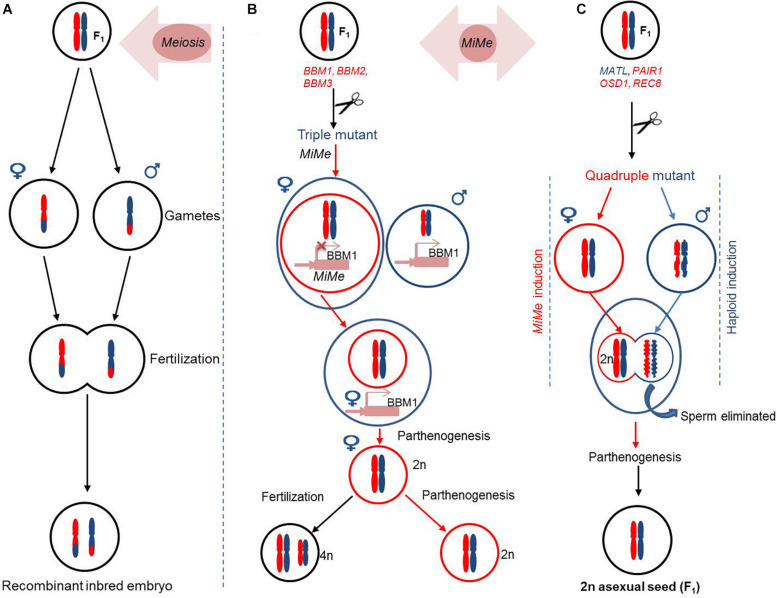
Work flow for synthetic apomixes in rice. **(A)** Normal meiosis process promotes segregation and produces recombinant inbred embryo. **(B)** Triple mutant of *BBM1*, *BBM2*, and *BBM3* triggers *MiMe* and causes embryo abortion during early fertilization in ovary, which can be rescued by male-transmitted *BBM1* to produce asexual 2n seeds through parthenogenesis. **(C)** Triple mutant of *PAIR1*, *OSD1*, and *REC8* promotes *MiMe* phenomenon to avoid fertilization. The *MATL* in quadruple mutant of *MATL*, *PAIR1*, *OSD1*, and *REC8* promotes haploid production by eliminating parental chromosomes during fertilization and facilitates parthenogenesis to produce 2n asexual clonal seeds. *MiMe*, mitosis instead of meiosis.

Hybrids are the ultimate source of improved production, but successive selfing and genetic segregation can induce inbreeding depression and the loss of hybrid vigor. During a normal meiotic process, the gametes undergo recombination, crossover, and fertilization to produce recombinant inbred plants (Figure 8A). This process, upon successive selfing, reduces heterosis. Hence, bypassing meiosis and fertilization could directly convert the gametes into adult plants that could be propagated through seeds without segregation in the successive generations through a phenomenon called apomixes. This can be achieved by modulating the postfertilization embryogenesis pathway. A genetic approach called “mitosis instead of meiosis” (*MiMe*) eliminates recombination by substituting mitosis for meiosis, producing clonal seeds through parthenogenesis (Marimuthu et al., 2011; Mieulet et al., 2016). Clonal propagation of F_1_ seeds through apomixes maintains the genome-wide heterozygosity in hybrid plants by avoiding segregation.

The *MATRILINEAL* (*MATL*; also known as NOT LIKE DAD and *PHOSPHOLIPASE A1* (*ZmPLA1)* is a pollen phospholipase gene that has been edited to produce haploid maize plants (Gilles et al., 2017). The *MATL* ortholog in rice is *OspPLAIIφ*, a gene that has a pollen-specific expression pattern in mature panicles before pollen shedding. Targeted CRISPR/Cas9-induced *OspPLAIIφ* mutations also resulted in haploid plants in rice that were able to produce normal pollen and seed setting at a low rate. Haploidy analysis revealed a haploid induction rate of up to 1.6% in the E_1_ population, which increased to 4.6% in the E_2_ population after outcrossing with other female tester lines. On average, the haploid induction rate through homozygous mutation in *OspPLAIIφ* was ∼6% with an average seed setting rate of up to 20%, which is similar to the results achieved in the maize *MATL*. This observation led to the renaming of *OspPLAIIφ* to *OsMATL* (Yao et al., 2018).

The *BABY BOOM1* (*BBM1*) gene, a member of the APETALA 2 transcription family, can trigger parthenogenesis through its ectopic expression and bypass fertilization in the female gamete. The triple *BBM1*, *BBM2*, and *BBM3* mutant promotes mitosis instead of meiosis (*MiMe*) and avoids fertilization, resulting in embryo abortion. This aberrant expression can be fully rescued by male transmitted *BBM1* (Figure 8B). The combined expression of *MiMe* and *BBM1* in the egg cells produces diploid maternal clones through parthenogenesis that are able to retain the F_1_ heterozygosity (Khanday et al., 2019). Despite the tremendous advantages of producing asexual lines, an apomictic lineage of *BABY BOOM* genes fosters side effects of polyploidy, which has always been a reproductive barrier through genetic self-incompatibility of the parental lines. The self-fertilization of the diploid clones leads to ploidy doubling in each generation, which should be prevented for true apomixes. The issue has recently been resolved by the introduction of *OsMATL* gene.

The issue has recently been resolved by the use of rice *OsMATL* gene. The editing of the pollen-specific rice gene *MATRILINEAL* (*OsMATL*) can produce haploid seeds from hybrid rice (Yao et al., 2018). Moreover, the triple mutant of the meiosis-specific genes *REC8*, *PAIR1*, and *OSD1* turns meiosis into mitosis (*MiMe*) during female embryogenesis and blocks fertilization to produce an apomictic embryo. Fixing the expression of the haploid inducer *OsMATL* and the *MiMe* inducers *REC8*, *PAIR1*, and *OSD1* through multiplex genome editing results in the elimination of sperm cells and produces diploid seeds by parthenogenesis (Figure 8C). The clonal plants can be propagated through these seeds without undergoing genetic segregation and the doubling of the ploidy level (Wang et al., 2019). This technique is therefore very efficient to engineer meiotic recombination for clonal hybrids with the desired heterozygosity. This mode of reproduction is advantageous compared to the two-line hybrid production scheme and is also less labor intensive. However, it abolishes genetic variation, as the resultant clonal embryos harbor complete maternal genetic complement.

### Engineering Meiotic Recombination for Reverse Breeding

Normal meiosis believes in crossover recombination, a crucial process in plant breeding to create novel allele combinations on chromosomes for breeding superior F_1_ hybrids. Silencing the crossover formation/homologous chromosomes pairing is essential for breeders to effectively engineer the chromosomes behavior for hybrid vigor (Wijnker and Jong, 2009). The F_1_ heterozygotes cannot be stably propagated through seeds, and establishing breeding lines for elite heterozygote has always been a major obstacle for plants breeders. Reverse breeding can efficiently develop the homozygous parental double haploid lines, which can stably be propagated indefinitely. These homozygous parental lines upon mating can then reconstitute the elite heterozygote. The technology is based on the silencing of meiosis-related target genes in heterozygote, which then produces reduced number of viable haploid. The perfectly homozygous double haploid plants can be regenerated form haploid spores (Dirks et al., 2009; Ren et al., 2017). The *Arabidopsis DMC1* has successfully been silenced to develop achiasmatic haploid and double haploid plants to reconstitute the heterozygote (Wijnker et al., 2012, 2014). Over the past decade many homologous recombination-related genes have been identified, but their potential for hybrid rice breeding has never been harvested. For example, the *PAIR2*, *PAIR3*, *BVF1*, and *SPO11* are essential for chromosome recombination, and the mutations cause complete male sterility (Nonomura et al., 2004; Yuan et al., 2009; Yu et al., 2010; Zhou et al., 2017). Interestingly, their mutants produce intact univalent, hence perfectly useful. These and the other related genes can be the soft target of knocking down the gene expression through RNAi to promote reverse breeding in rice in the future. However, chromosome recombination can also be engineered to increase crossover incidences and position over chromosomes to develop F_1_ hybrids with maximum heterozygosity.

## Conclusion and Perspectives

Male sterility is an inevitable phenomenon that has long been exploited to enhance rice productivity. Over the last decade, many GMS genes have been identified and their regulatory pathways investigated, even though further studies are needed to explore in more detail genetic and molecular mechanisms in order to avoid the genetic vulnerability of hybrids. The use of advanced biotechnological tools and comparative functional genomics will very likely result in gains in the field of male sterility and hybrid rice breeding. Comparative functional genomics analyses can greatly assist in the identification of putative GMS genes in coordination with functional complementation studies (Fernandez Gómez and Wilson, 2014). In this review, we have summarized and illustrated the different genetic, biochemical, and molecular mechanisms that might prove crucial in determining male fertility in rice.

While different genetic and functional genomics studies have broaden our understandings about male sterility regulation and the application of different technologies to the utilization of heterosis, a few important questions remain unanswered and require further investigation, specifically: (1) What is the mechanism of stamen primordial formation and what kind of genetic mechanisms are involved in this process? (2) What is the metabolic contribution of the anther epidermis and endothecium during pollen development in rice? (3) What strategies are needed to buffer the adverse effects of sudden environmental fluctuations on EGMS line breeding? Although many new breeding techniques have been developed during the last decade, there are still some aspects that always remained unnoticed. For example, the rice epigenetic landscape is an excellent platform to create transcriptional and posttranscriptional gene silencing without disruption in genomic sequences. However, to date, no epigenetic genome modifications/silencing have been reported to improve crop productivity. Recently, many new plant breeding techniques, including RNA-directed DNA methylation (RdDM), have been suggested (Schaart et al., 2016). RdDM is based on the generation of small interference RNAs (21–24nts) and also suppresses TEs. The epigenetic modifications through RdDM are inherited to generations; hence the technique can accelerate crop improvement by producing transgene-free plants (Wakasa et al., 2018). In addition, the autotetraploid rice is a rich germplasm resource, but hybrid sterility has been the limiting factor for their application in hybrid rice breeding. Recently, some studies have analyzed the important contribution of meiosis-related genes (*OsMND1*) toward increased performance of autotetraploid rice hybrids (Xiong et al., 2019; Koide et al., 2020). In addition, it has been recently identified that polyploidy (autotetraploid and neo-tetraploid) rice hybrids exhibited higher expressions of several genes involved in carbohydrate and lipid metabolism during pollen development and displayed higher seed setting and positive heterosis as compare to parental lines (Chen et al., 2019; Ghaleb et al., 2020). The combination of genomics analysis to identify high seed fertility-related genes and genome editing techniques is needed to harvest the potential usefulness of autotetraploid and neo-tetraploid germplasm for rice breeding in future.

A proper manipulation of male fertility genes would be effective for hybrid production in rice. During the developmental process of the anther and pollen, genes related to lipid metabolism usually show resistance against biotic and abiotic stresses (Yeats and Rose, 2013). Therefore, these genes should be a priority to facilitate hybrid breeding. Very recently, a maize male fertility gene (*ZmMS26*) was targeted by the CRISPR/Cas system to produce a one-step hybrid (Qi et al., 2020). Similarly, another lipid-metabolism-related maize gene *ZmMs17* has recently been used to create male sterility in rice, and interestingly, it did not affect the vegetative growth and female fertility (An et al., 2020). Importantly, rice also contains *ZmMs17* and *ZmMS26*-like genes, specifically *PTC1/OsMS1* and *CYP704B*, respectively, whereby this technology can be readily applied to these genes in rice plants, along with other fertility-determining genes in order to establish and develop stable hybrid rice seeds in future. Controlling the development of pollen for selective hybrid breeding has become a major strategy, but importantly, this should not be detrimental to the vegetative growth of the plant. A careful analysis of male sterile parents for the production of hybrids is thus very important before their commercial application is implemented. Similarly, the prediction of hybrids with a superior performance should be conducted before commercial release. Hence, it would be helpful to estimate hybrid potential by conducting genomic selection of hybrids using single nucleotide polymorphism (SNP) or insertion deletion (InDel) genotyping and evaluating their phenotypic performance against different agronomic traits of interest (Cui et al., 2020; Guttikonda et al., 2020).

This review provided a detailed evaluation on how different effective strategies, including the integration of male sterility related genes, functional genomics analyses, and biotechnological tools, need to be combined to ensure a stable hybrid production and global food security.

## Author Contributions

AA, PY, SC, and LC jointly designed the idea and writing structure of the manuscript. AA and PY wrote the manuscript including all figures and tables. LC and SC approved and designed the outlines of the manuscript. ZY, LS, and DC contributed to the revision and provided critical feedback. All the authors contributed to the article and approved the submitted version.

## Conflict of Interest

The authors declare that the research was conducted in the absence of any commercial or financial relationships that could be construed as a potential conflict of interest.

## Footnotes

1 http://ricexpro.dna.affrc.go.jp/
